# Human DNA hijacking microbiota surveys: causes and consequences in colon related 16s rRNA amplicon sequencing

**DOI:** 10.1017/gmb.2025.10012

**Published:** 2025-08-19

**Authors:** Leandro Di Gloria, Lorenzo Casbarra, Marta Bastiani, Gabriele Memoli, Matteo Ramazzotti

**Affiliations:** Department of Experimental and Clinical Biomedical Sciences, University of Florence, Florence, Italy

**Keywords:** microbiota, low biomass, sequencing, off-target, mis-priming, host, human

## Abstract

The efficiency of polymerase chain reaction (PCR) decreases under suboptimal conditions, such as low template concentration combined with high concentrations of similar sequences. Under these circumstances, mis-priming can occur, leading to the generation of erroneous copies. Specifically, in 16S amplicon sequencing of human intestinal biopsy samples, host off-target sequences are frequently generated and subsequently sequenced, particularly when the commonly used V3-V4 primers are employed. This issue not only introduces errors in data interpretation but also results in the unnecessary consumption of sequencing depth. In response to this challenge, we analysed over 1,300 publicly available V3-V4 amplicon sequences related to the human colon, profiling the colon microbiota while elucidating the biases introduced by host off-targets. Briefly, our findings reveal that unaddressed host DNA contamination can lead to false bacterial identifications and obscure significant differences in microbiota composition. Furthermore, we identified human sequences on chromosomes 5, 11, and 17 as the main contributors to the majority of off-target sequences. Finally, we suggest practical approaches to mitigate this issue without altering the original protocol design, retaining the widely used V3–V4 primers. In particular, using a C3 spacer-modified nucleotide targeting the off-target sequence is here proposed as a promising strategy acting upstream of the off-target generation.

## Graphical abstract

**Figure:**
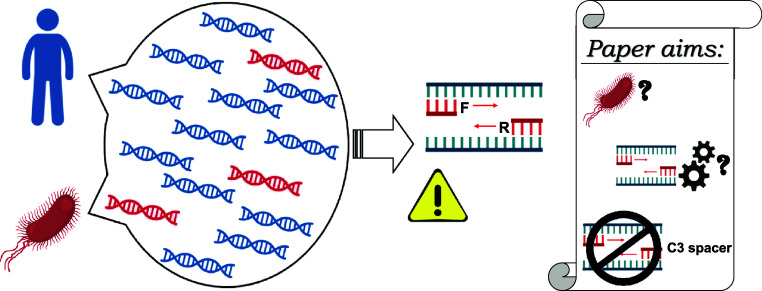

## Highlights

- Host DNA off-targets bias microbiota profiling through 16S V3–V4 sequencing;
- Most off-targets are caused by mis-priming to human chromosomes 5, 11, and 17 loci;
- Most off-targets share a 5′ motif and are approximately 300 bp in length;
- Off-target formation is preventable or, at a minimum, effectively mitigable.

## Introduction

The invention of polymerase chain reaction (PCR) in 1983 by Kary Mullis (Mullis, 1990) revolutionised molecular biology, enabling diverse methodologies for amplifying, selecting, and modifying nucleic acid sequences. For instance, microbial communities can be studied by sequencing the amplified hypervariable regions of the 16S rRNA gene, rather than whole genomes (Sipos et al., 2007; Bacci et al., 2025). This is achieved through the use of degenerate primers designed to anneal to conserved “universal” regions flanking the hypervariable segments, ensuring the inclusion of the largest diversity of prokaryotes (Sipos et al., 2007; Bacci et al., 2025). 16S rRNA amplicon sequencing significantly reduces the depth of sequencing required, thereby allowing more samples to be processed within a given experimental budget. Over recent decades, the collective effort of numerous researchers has uncovered much of the human gut microbiota by combining the high throughput of next-generation sequencing (NGS) with 16S amplicon sequencing. For example, diverse authors highlighted significant differences in the microbiota of subjects suffering diseases such as IBD (intestinal bowel disease) (Kamada et al., 2013; Ryan et al., 2020), chronic chough (Baldi et al., 2024) and CRC (colon rectal cancer) (Saffarian et al., 2019; Borgognone et al., 2021; Choi et al., 2021; Debelius et al., 2023; Russo et al., 2023). The gut microbiota profiling often relies on the collection of patient faeces rather than colon tissue, as faecal samples are generally representative of the colon microbiota composition (Russo et al., 2023) while minimizing ethical concerns associated with sample collection. However, many researchers prefer to collect actual colon tissue samples whenever feasible, as faecal and colon tissue samples are not identical despite their similarities (Russo et al., 2023).

Technical challenges limit the effectiveness of NGS and amplicon sequencing when working with colon biopsies due to their low microbial biomass relative to the total amount of host DNA. This results in artifacts such as the increased influence of “kitome” sequences (ubiquitous kit contaminants) (Paniagua Voirol et al., 2021; Di Gloria et al., 2024; Bacci et al., 2025) and the host DNA “off-target” amplifications (Walker et al., 2020; Mayer et al., 2021). The latter issue may be unexpected due to the use of primers designed to amplify bacterial sequences and may severely hamper the analysis. For example, in 2021 Bedarf et al. evidenced that host off-targets misclassified as bacteria led to false positive bacterial detection in brain tissues, calling into question the recent discoveries regarding the brain microbiome (Bedarf et al., 2021). In fact, several studies focused on human tissues related microbiota did not perform off-target decontaminations, at least not explicitly (Lu et al., 2016; Saffarian et al., 2019; Ryan et al., 2020; Bedarf et al., 2021; Borgognone et al., 2021; Liu et al., 2021). The off-targets induced biases may be mitigated in silico by aligning the reads to the human genome through tools such as Bowtie2 (Langmead & Salzberg, 2012; Bharti & Grimm, 2019) and thereby removing them. However, identifying and removing the contaminants after the sequencing inevitably results in wasting sample sequences, undermining the cost-efficiency of the 16S amplicon sequencing approach and reducing the estimated alpha diversity (Mayer et al., 2021). The use of primers targeting the V1-V2 regions of the 16S rRNA gene has been proposed as a strategy to significantly reduce the generation of host off-targets, in contrast to the more commonly employed V3-V4 regions (Walker et al., 2020; Bacci et al., 2025). However, focusing on the V1-V2 region may result in the underrepresentation of archaea (Bharti & Grimm, 2019; Abellan-Schneyder et al., 2021) and certain taxa as *Prevotella, Streptococcus*, and *Fusobacterium* (Deissová et al., 2023). This difference is noteworthy, especially when the CRC microbiota is analysed, given that *Fusobacterium nucleatum* strains are well-known contributors to such disease (Rubinstein et al., 2013).

The off-target amplifications are also observed when the opposite imbalance occurs, where human DNA serves as the primary template and microbial DNA causes off-target amplified sequences, such as in forensic analyses (Bokulich et al., 2013). In fact, although the efficiency of the PCR hinges on the precise annealing of complementary sequences, namely the primers and the template, this biological event is still driven by measurable probabilities (Boyle et al., 2009). Accordingly, the mis-priming (annealing of not perfectly complementary sequences) (Boyle et al., 2009) is more likely to occur under certain conditions. Such probabilities depend on various factors including the annealing temperature (Rychlik et al., 1990; Sipos et al., 2007; Boyle et al., 2009), high G–C in 3′ ends of primers which causes this end to act as primer even in absence of the 5′ annealing (Rychlik, 1995), the nucleotides involved in the mismatch and their position toward the primer’s 5′ or 3′ (Kwok et al., 1990), chemical characteristics of the PCR buffer, template concentration (Rychlik et al., 1990; Sipos et al., 2007) and, crucially, the design of the primers sequence. Conversely, many of these parameters have already been established by the official 16S amplicon sequencing Illumina protocol (Illumina n.d.). Implementing alternative PCR conditions could impede efforts to achieve the standardization that microbial ecology urgently requires. In light of this, this study primarily aimed at investigating the biases introduced by host off-targets in human colon microbiome analysis and to elucidate the underlying reasons for their occurrence, with the goal of proposing direct solutions or, at the very least, paving the way for future improvements. Indeed, identifying the sequences where mis-priming occurs would enable the development of strategies to reduce their amplification, such as the use of C3 spacer-modified nucleotides targeting the off-target sequences, as previously proposed (Vestheim & Jarman, 2008) and tested in this paper.

In addition, a detailed meta-analysis on over 1300 downloaded samples with a similar experiment design was conducted to support the characterization colon microbiome.

## Methods

### 16S sequences collection from NCBI database

A total of 1,357 samples obtained through V3–V4 amplicon sequencing of DNA extracted from human colon biopsies were retrieved from the NCBI Sequence Read Archive (SRA). These samples are distributed across ten bioprojects (Table 1), among which eight used the primer pairs 341F and 805R, and two (PRJNA298957 and PRJNA325650) used the pair 338F and 806R. Notably, the methods section of bioproject PRJNA325650 refers to the forward primer as “319F,” but its sequence actually corresponds to 338F. Sequences derived from tissues preserved in paraffin were excluded from the analysis due to the potential for paraffin-induced single-nucleotide mutations (Di Gloria & Niccolai, 2022), which would strongly undermine the objectives of this research. Moreover, we preferred to focus on DNA derived from directly processed samples rather than the paired frozen biomasses, when such information was reported in the bioproject. The primary characteristics of each bioproject are listed in Table 1.

**Table 1.** List of the V3-V4 16S colon microbiota sourced amplicon datasets analysed in this study

References	SRA BioProject	Kit used for DNA extraction	Collected tissue	Focus	Sequences downloaded
Russo et al. (2023)	PRJNA899104	DNeasy PowerSoil	Mucosa	CRC (samples from saliva, stool, and colon)	53
Ryan et al. (2020)	PRJNA398187	AllPrep DNA/RNA Mini kit	Mucosa	IBD, Healthy tissues	371
Choi et al. (2021)	PRJNA743150	Unspecified	Unspecified	CRC, Healthy tissues	104
Borgognone et al. (2021)	PRJEB46353	DNeasy Blood and Tissue	Mucosa	CRC (comparison between frozen samples and paraffin)	10
Debelius et al. (2023)	PRJEB57580	ZR–96 Genomic DNA MagPrep	Unspecified	CRC, Healthy tissues	203
Saffarian et al. (2019)	PRJNA507548	PicoPure DNA extraction	Mucosa and Crypt	CRC, healthy tissues, healthy subjects	229
Park et al. (2024)	PRJNA995580	Chemical	Mucosa	CRC, healthy tissues	230
Drewes et al. (2017)	PRJNA325650	MasterPure DNA Purification Kit	Mucosa	CRC, healthy tissues	51
Niccolai et al. (2022)	PRJNA840857	DNeasy PowerSoil	Mucosa	Churg–Strauss	7
Lu et al. (2016)	PRJNA298957	E. Z. N. A.® Soil DNA Kit	Mucosa	CRC, healthy tissues, healthy subjects	99

*Note*: The term “Unspecified” indicates that the related information was not explicitly stated in the publication associated with the dataset.

Furthermore, the obtained results regarding the host off-targets were tested on 130 additional samples from three datasets (PRJNA877831 (Niccolai et al., 2023), PRJNA835844 (Curini et al., 2023), PRJNA678413 (Liu et al., 2021). These bioprojects are also related to V3-V4 sequencing of DNA of human tissues but are not specific to the colon biopsies, as they are focused, respectively, on breast, aortic valve, and gastric biopsies.

### Microbiota analysis and off-target related bias estimation

Demultiplexed sequence reads were processed using QIIME2 (Bolyen et al., 2019) version 2024.2. Sequencing primers and reads without primers were removed using the Cutadapt tool (Martin, 2011). DADA2 (Callahan et al., 2016) was used to perform the filtering, merging, and chimera removal steps of the paired-end reads after cutting low-quality nucleotides from both forward and reverse reads. The DADA2 settings were customized individually, tailored to the specific base qualities of each bioproject. The ASVs (amplicon sequence variants) were then generated and aligned to the human genome reference “T2T - CHM13 v.2” (Nurk et al., 2022) using Bowtie2 (Langmead & Salzberg, 2012) to outline the off-target sequences. Subsequently, the ASVs were divided into two separate datasets: one containing off-target-contaminated ASVs and the other consisting of decontaminated ASVs. Both datasets were processed independently using the same analytical pipeline detailed below, in order to compare the results obtained. The taxonomic assignments were performed using the Scikit-learn naive Bayes multinomial classifier re-trained on the SILVA SSU database (Release 138) V3-V4 hypervariable database. All ASVs associated with genera with the highest relative abundance in samples below 0.01% cut-off or featured in less than 1% of the samples were discarded to minimize contaminants and sequencing errors and to improve statistical inference (Bokulich et al., 2013) (Cao et al., 2020). Every read not identified at the phylum level or identified as from mitochondria or chloroplast was discarded.

Subsequently, the statistical analyses on bacterial communities were performed in R 4.3 using the packages phyloseq 1.44.0 (McMurdie & Holmes, 2013), vegan 2.6–4, and other packages satisfying their dependencies. The packages ggplot2 3.4.2, ggh4x 0.2.4, and ggpubr 0.6.0 were used to plot data and results. A rarefaction analysis on genera was performed on every sample using the function rare curve (step 100 reads), further processed to highlight saturated samples (arbitrarily defined as samples with a final slope in the rarefaction curve with an increment in genus number per reads <1e−4). The unsaturated samples were used to investigate the off-targets but were not included in the microbiota analysis. The most abundant phyla and genera were identified according to their highest average relative abundance among the samples. Principal coordinate analysis (PCoA) was performed at the genus level using the Hellinger distance (Euclidean distance on Hellinger transformed abundances), given the sparse and compositional properties of the data (Legendre & Legendre, 2012; Yerke et al., 2024).

To further explore the bias introduced by the off-targets on the statistical analyses, the differential abundances between CRC tissues and healthy tissues were inferred using the R package DESeq2 1.42.0 (Love et al., 2014). This comparison was specifically chosen due to the large number of samples available and to include subject ID in the statistical design, thereby compensating for variability between subjects and across different studies. The samples related to IBD tissues and to the paired healthy tissues were not included in this comparison to simulate a proper research question, namely, the difference in taxa abundances between CRC tissues and healthy tissues. The analysis was conducted on both the off-target-contaminated and decontaminated datasets, and the results were subsequently compared. Every DESeq2 result with *p*-value (adjusted with Benjamini–Hochberg method) lower than 0.05, log2FoldChange greater than 1, and base-mean value greater than 50 was considered significant.

The bash script used to download and process the 16S raw FASTQ and the R script of the microbiota analysis and tables with further details are publicly available at the link https://github.com/LeandroD94/Papers/tree/main/2025_Host_OffTargets_16SHumanBiopsies.

### Investigating the causes of off-target generation

The SAM files generated by aligning the ASVs to the T2T-CHM13 v2 reference genome using Bowtie2 were analysed to quantify off-target incidences across chromosomes and determine their exact mapping positions. Subsequently, the sequences of the most prevalent off-targets were aligned to identify consensus motifs. The R packages **DECIPHER** v 2.30 (Wright, 2016) and **ggseqlogo** v 0.2 were employed to align the most prevalent off-targets and represent the consensus, respectively. The bash and R scripts employed are publicly accessible at the link above.

Furthermore, the resulting consensus sequence was queried against the human genome reference T2T-CHM13 v2 using NCBI BLASTn (Camacho et al., 2009) optimized on the *Homo sapiens* genome (https://blast.ncbi.nlm.nih.gov/Blast.cgi?PAGE_TYPE=BlastSearch&BLAST_SPEC=OGP__9606__9558&LINK_LOC=blasthome). Finally, BLASTn searches of the consensus sequence were performed also on the nucleotide collection (nr/nt) database, restricted to bacterial and archaeal sequences, to identify eventual prokaryotic sequences exhibiting similarities with the identified consensus.

### Testing the amplification of off-targets DNA

Real-time PCRs were performed to validate the bioinformatics predictions. The QIAamp PowerFecal Pro DNA® Kit (Qiagen, Hilden, Germany) was used to extract DNA from Hep G2 cell lines (human hepatocarcinoma cells), cultured in Dulbecco’s Modified Eagle Medium (DMEM) supplemented with 10% fetal bovine serum (FBS), penicillin, and streptomycin. The absence of *Mycoplasma* in Hep G2 cultures was confirmed through the Mycoplasma PCR Detection® kit (BioVision Inc., United States). Additionally, the DNA was extracted from the faecal sample of a healthy individual, serving as high microbial biomass matrix, hence acting as a positive control. The extracted DNA was used as input for PCR reactions performed with the SsoAdvanced™ Universal SYBR® kit (BioRad, United States) and the CFX96 Real-Time PCR Detection System (BioRad, United States) termocicler. A negative control of both the DNA extraction kit and the PCR kit was included in the experiment. The “first PCR” was designed according to Illumina’s standards **(Illumina)**, consisting in 25 cycles on a negative control, 12.5 ng of positive control DNA and 12.5 ng of human DNA, using the 341F (CCTACGGGNGGCWGCAG) and 805R (GACTACNVGGGTWTCTAATCC) prokaryotic primer pair addressing the V3-V4 region of the 16S gene (Albertsen et al., 2015). Specifically, for the negative control, the sample itself was added to the final reaction volume, as no DNA was detected based on quantification and the 260/280 nm ratio via spectrophotometry. The “second PCR” was designed with the goal of enhancing contaminant formation for study purposes; hence, it consisted of 35 cycles and the amount of template DNA was increased to 50 ng (which is *the* limit of the employed PCR kit) in case of the negative control and the samples involving human DNA. The nature and the amount of the template as well as the primers employed for each tube in the second PCR, are reported in Table 2. The “third PCR” consisted of 35 cycles and was conducted on human DNA amplified with the prokaryotic 341F and 805R primer pair and increasing concentration of the custom off-target inhibitor oligonucleotide sequence (Table 2).

**Table 2.** Overview of the three PCR batches, detailing the samples and experimental settings

Assay number	Sample	Template amount	Cycles	Primer F	Primer R	Inhibitor
1	Negative control	up to VF	25	341F (0.5 μM)	805R (0.5 μM)	none
1	Faeces DNA (positive control)	12.5 ng	25	341F (0.5 μM)	805R (0.5 μM)	none
1	Human DNA	12.5 ng	25	341F (0.5 μM)	805R (0.5 μM)	none
2	Negative	up to VF	35	341F (0.5 μM)	805R (0.5 μM)	none
2	Faeces DNA (positive control)	12.5 ng	35	341F (0.5 μM)	805R (0.5 μM)	none
2	Faeces DNA	12.5 ng	35	5 cons (0.5 μM)	805R (0.5 μM)	none
2	Human DNA	50 ng	35	5 cons (0.5 μM)	805R (0.5 μM)	none
2	Human DNA	50 ng	35	341F (0.5 μM)	805R (0.5 μM)	none
2	Human DNA	50 ng	35	341F (0.5 μM)	805R (0.5 μM)	none
2	Human DNA	50 ng	35	341F (0.5 μM)	805R (0.5 μM)	none
2	Human DNA	50 ng	35	341F (0.5 μM)	805R (0.5 μM)	0.25 μM
2	Human DNA	50 ng	35	341F (0.5 μM)	805R (0.5 μM)	0.5 μM
3	Human DNA	50 ng	35	341F (0.5 μM)	805R (0.5 μM)	0.25 μM
3	Human DNA	50 ng	35	341F (0.5 μM)	805R (0.5 μM)	0.5 μM
3	Human DNA	50 ng	35	341F (0.5 μM)	805R (0.5 μM)	1 μM

*Note*: Concentrations are reported in molarity (M). The term ‘5’ cons’ refers to the oligonucleotide designed based on the off-target 5′ consensus and used as a forward primer, while ‘inhibitor’ refers to the same oligonucleotide modified with a 3’ C3-spacer.

The human sequence 5’-TGATAAACCTTTAGCAATAAACSA-3′ (“5 cons” in the Table 2), defined as the 5′ off-target’s consensus according to the bioinformatics observations in this paper, was used as forward, along with the prokaryotic 805R as reverse in certain samples. It is noteworthy that this sequence was designed to anneal to the human genome immediately downstream of the region resembling the prokaryotic forward primer. The online tool Oligoanalyzer (https://www.idtdna.com/pages/tools/oligoanalyzer) was used to confirm that the consensus sequence does not form hairpins and has a melting temperature comparable to that of the prokaryotic primers. Moreover, the same human sequence modified with the addition of a 3’ C3 spacer (hence acting as a potential “inhibitor” if annealed) was used in additional reactions employing the 341F and 805R pair to further test its recurrence in the 16S primers off-target amplicons.

The amplicon sizes were assessed by electrophoresis, loading 1 μL of each PCR product on a 2% agarose gel run for 45 minutes and stained with ethidium bromide. The GeneRuler 100 bp Plus DNA Ladder (Thermo Fisher Scientific, United States) was run alongside the samples as a molecular weight marker.

## Results

### Host target induced biases in sequence processing

A total of 110,425,198 sequences downloaded from NCBI were processed, among which 48,166,716 (43.6%) passed every quality filter. In particular, approximately 50% of the raw sequences were removed due to quality issues, while an additional 3% were excluded after being identified as host off-targets. About 3% were discarded based on abundance and prevalence thresholds or being identified as non-prokaryotic (chloroplasts, mitochondria, and sequences unidentified at phylum level). The bioprojects displayed varying levels of host off-targets, ranging from less than 0.1% (PRJNA840857) to almost 30% (PRJNA743150) of the reads passing the quality filters (Supplementary Figure S1). Although the two bioprojects using primer 338F instead of 341F (PRJNA298957 and PRJNA325650) were among those with fewer off-targets, another project employing primer 341F, namely PRJNA840857, showed an even lower amount. Notably, the taxonomic classification required manual specification of the read orientation (5′ → 3′) as the classifier used would otherwise automatically and silently assume an incorrect orientation in a few bioprojects. Twenty-five samples were discarded as they were deemed unsaturated, indicating that additional sequencing depth was required to accurately represent the microbial community. Of these, 23 samples belonged to bioproject PRJNA507548, which had 14% of its reads classified as off-targets. Moreover, 17 of the unsaturated samples were derived from paired CRC tissues or their corresponding healthy counterparts, resulting in additional samples being excluded during the paired comparison analysis. Also, the unsaturation observed in only a subset of samples within a bioproject indicates variability in off-target occurrence even among samples from the same batch.

### Host-target induced biases on microbiota analysis

**Figure 1.**
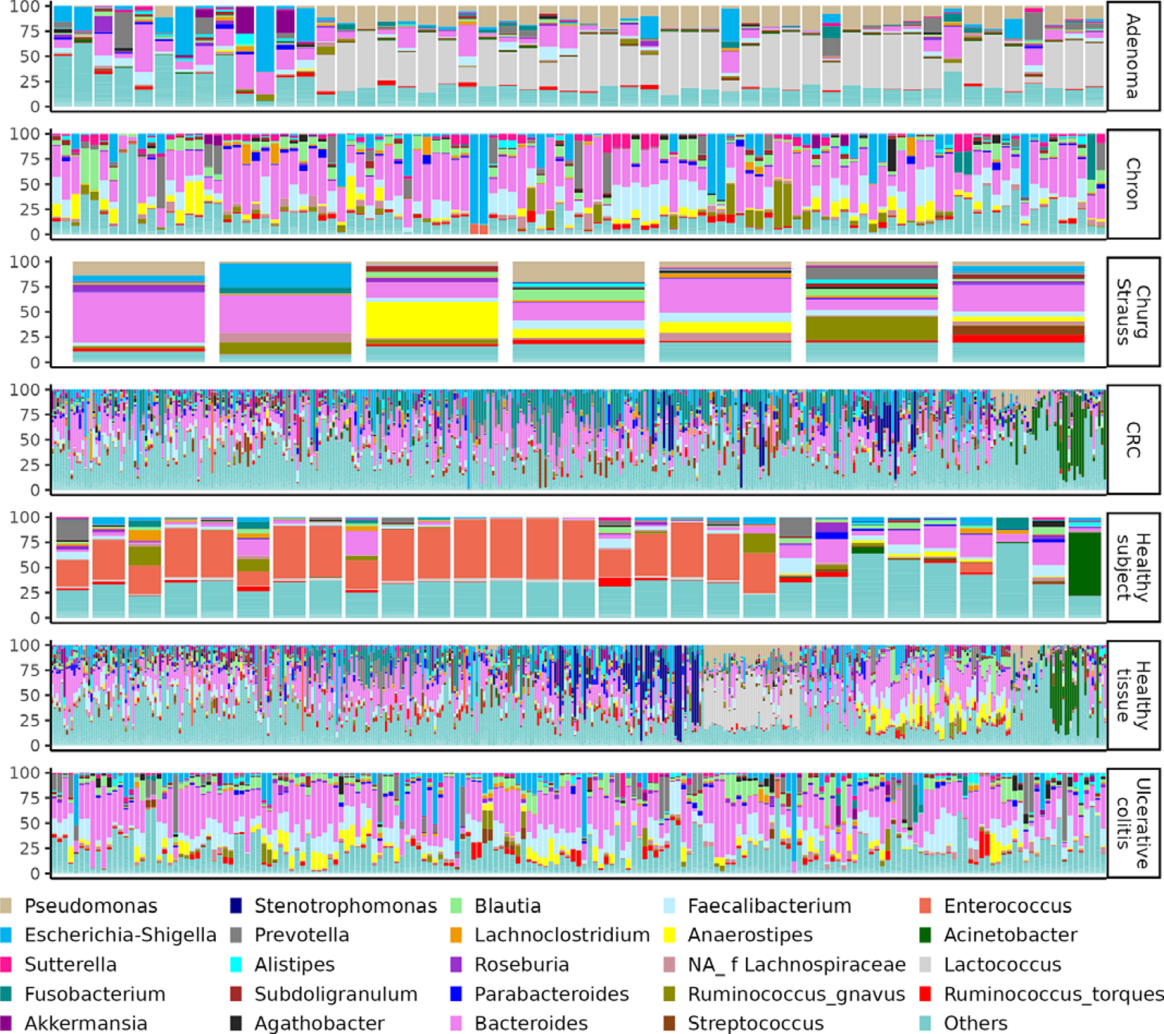
Bar plot representing the percentage abundances of the 24 most abundant genera across every analysed colon related bioproject. Each vertical bar represents the abundances within a sample. The samples names (along the X axes) were not included in the plot due to visibility issue caused by the high number of samples. The samples are clustered according to their nature or patient disease.

The 24 most abundant genera represent most of the colon sample compositions, with the exclusion of few healthy tissues, which were characterised mostly by other genera (Figure 1 and Table 3). Notably, certain taxa such as *Enterococcus, Parabacteroides*, and *Acinetobacter* were predominant in many samples yet completely absent in others within the same sample group, suggesting a pronounced batch-induced bias across bioprojects, despite the uniform bioinformatics framework of this meta-analysis.

**Table 3.** Percentage abundances of the most abundant genera across every analysed colon related bioproject

Family	Genus	Average across samples	Average including off-targets
Bacteroidaceae	*Bacteroides*	19.40	17.88
Ruminococcaceae	*Faecalibacterium*	6.45	6.21
Enterobacteriaceae	*Escherichia-Shigella*	5.48	4.99
Fusobacteriaceae	*Fusobacterium*	4.73	4.24
Prevotellaceae	*Prevotella*	3.56	3.15
Lachnospiraceae	*Blautia*	2.9	2.77
Streptococcaceae	*Lactococcus*	2.67	2.63
Xanthomonadaceae	*Stenotrophomonas*	1.93	1.91
Pseudomonadaceae	*Pseudomonas*	1.84	1.7
Lachnospiraceae	*Anaerostipes*	1.83	1.79
Moraxellaceae	*Acinetobacter*	1.76	1.59
Streptococcaceae	*Streptococcus*	1.63	1.51
Tannerellaceae	*Parabacteroides*	1.57	1.48
Lachnospiraceae	*Ruminococcus torques* group	1.53	1.41
Rikenellaceae	*Alistipes*	1.42	1.38
Akkermansiaceae	*Akkermansia*	1.33	1.3
Lachnospiraceae	NA	1.3	1.25
Sutterellaceae	*Sutterella*	1.25	1.16
Lachnospiraceae	*Lachnoclostridium*	1.2	1.14
Enterococcaceae	*Enterococcus*	1.19	1.08
Ruminococcaceae	*Subdoligranulum*	1.07	1.04
Lachnospiraceae	*Ruminococcus gnavus* group	1.03	0.98
Lachnospiraceae	*Roseburia*	1	0.96
Lachnospiraceae	*Agathobacter*	0.92	0.87

*Note*: The column “Average across samples” reports the average computed in the off-target decontaminated dataset, while the column “Average including off-targets” shows the averages in the dataset including the off-targets ASVs.

As observable in Table 3, the averages of these genera would have been slightly biased if the host off-targets would have been maintained in the dataset. For example, the average percent abundance of *Bacteroides* and *Fusobacterium* would decrease from 19.40% and 4.73% to 17.88% and 4.24%, respectively, if the off-targets were not removed.

Additional genera were identified exclusively in the off-target-contaminated dataset, namely *Opalinata* (a protozoan), *AKIW781*, *RBG-13-54-9*, *Candidatus* Uhrbacteria, and *Anaerosalibacter.* Their absence in the decontaminated dataset indicates that these are false positives arising from host DNA contamination.

PCoAs revealed a similar profile across many samples, forming a “core” in which numerous samples cluster, regardless of their bioproject or subject condition (Supplementary Figure S2). A few samples deviate from this core, displaying trends that appear to be influenced either by the bioproject batch (Supplementary Figure S3) or being related to CRC tissues and corresponding healthy tissues (Supplementary Figure S2). The presence of off-targets would have obscured this latter observation, as many samples from the bioproject PRJNA298957 (sequencing of biopsies from adenoma and healthy tissues, located in the lower portion of the PCoA plot) would have appeared farther from the other samples of CRC-related projects, exacerbating the batch effect-induced bias. Overall, the dataset including off-targets (Supplementary Figure S2 A) exhibits slightly greater variation along both PC1 and PC2 compared to the off-target-decontaminated dataset (Supplementary Figure S2 B), in addition to increased sample dispersion observed in some projects (Supplementary Figure S3). However, the biases introduced by off-target sequences were not substantial enough to obscure either the main differences between the microbiota of CRC tissues and their corresponding healthy tissues.

Finally, the influence of off-targets on the differential analysis was tested. The employed algorithm revealed significantly higher abundance of *Fusobacterium* and *Campylobacter* in CRC tissues compared to their paired healthy counterparts, whereas *Blautia* was more abundant in healthy tissues (Supplementary Figure S4). Notably, these differences would not have resulted in significant differences if the host off-targets had been retained in the dataset.

### Investigating the causes of off-target generation

**Figure 2.**
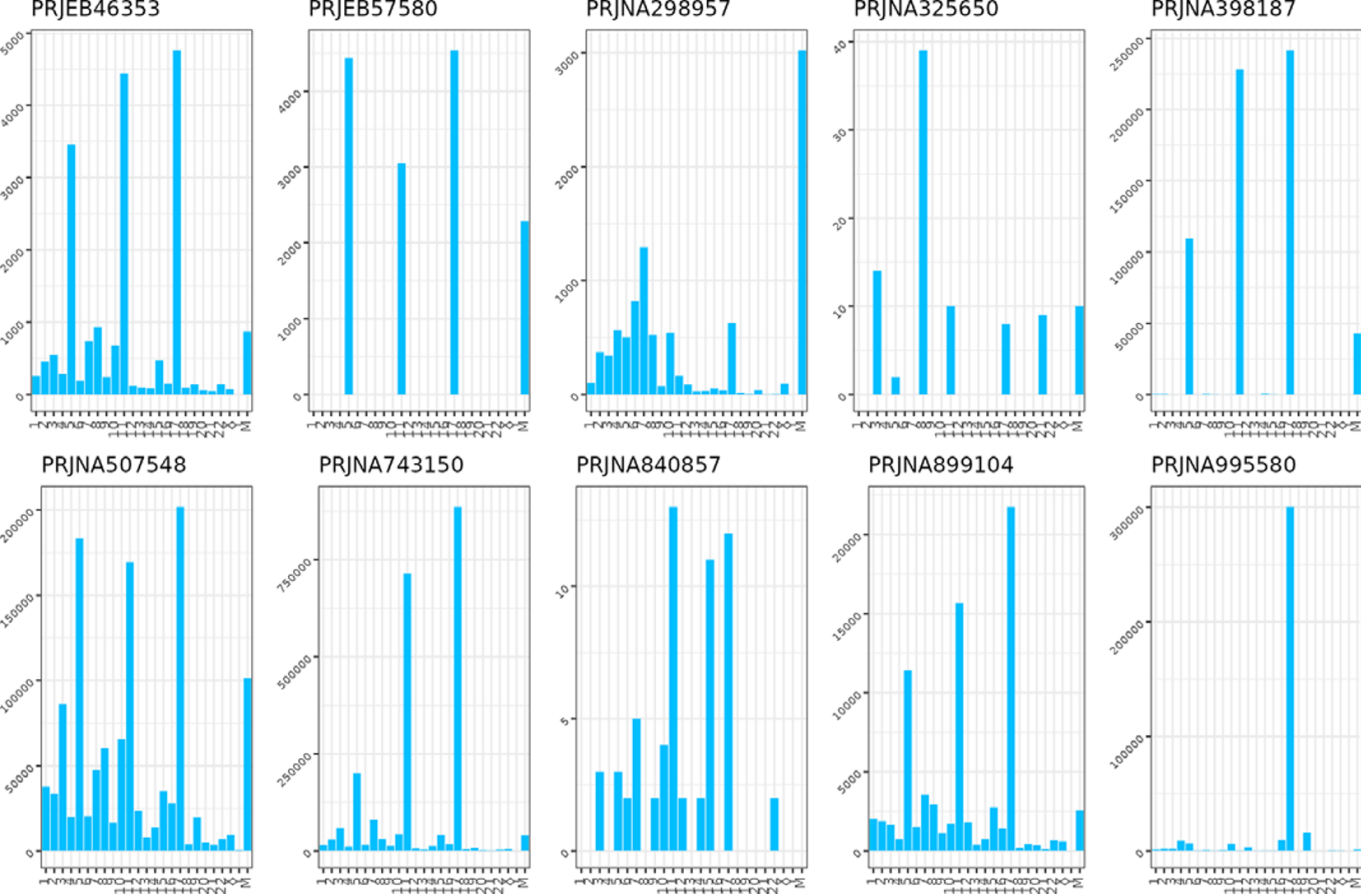
Bar plots showing the off-target reads total count (Y axis) across the bioprojects, clustered according to the human chromosome of origin (X axis).

The ASVs identified as originating from host DNA were distributed across multiple chromosomes in various bioprojects. Notably, a considerable proportion of these ASVs were aligned to chromosomes 5, 11, and 17, with the exclusion of the bioprojects PRJNA298957 and PRJNA325650 employing the primer 338F (Figure 2). Furthermore, the mitochondrial genome was a recurrent hit of such alignment, as in most datasets, the mitochondrial reads were among the most abundant contaminants following those from the aforementioned human chromosomes. An exception was observed only in the bioproject 298957, where mitochondrial sequences constituted the predominant source of contamination.

The 5′ ends of most of the ASVs originating from chromosomes 5, 11, and 17 were aligned to recurrent positions on these chromosomes (Supplementary Figures S5, S6, and S7, respectively) within each bioproject, including those employing the primer 338F. In particular, the consensus was found on chromosome 5 on the negative strand at position 81136755, on chromosome 11 on the negative strand at position 10594491, and on chromosome 17 on the positive strand at position 23,210,489 (according to the CHM13 reference available in NCBI GenBank) (Supplementary Figures S5, S6, and S7). The ASVs aligning to chromosome 17 emerged as the most abundant off-targets recurrently observed across bioprojects. Interestingly, in bioproject PRJNA995580, the chromosome 17-related off-targets aligned to a different position, a unique characteristic not observed in the other bioprojects (Supplementary Figure S7). The frequent off-target alignment to the same recurrent positions of chromosomes 5, 11, and 17, followed in prevalence by alignments to the mitochondrial genome, was also observed in the three additional bioprojects concerning samples not sourced from colon tissues (Supplementary Figure S8).

**Figure 3.**
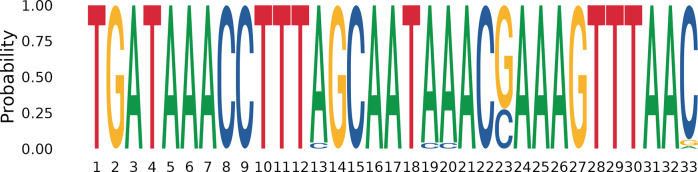
Logo of the consensus delineated at the 5′ end of the most abundant and recurrent off-target ASVs, excluding the forward primer sequence.

When the most abundant off-target sequences from each bioproject are aligned, the 32-nucleotide consensus sequence TGATAAACCTTTMGCAATMMACSAAAGTTTAAV can be identified at their 5′ end (Figure 3). If we exclude the degenerations involving nucleotides with extremely low frequency, the consensus sequence is TGATAAACCTTTAGCAATAAACSAAAGTTTAA. Notably, this 5′ end consensus is located immediately after the 3′ end of the forward primer in the ASVs (whose sequence is not included in the reported consensus).

As expected, numerous perfect hits (100% identity and 100% query coverage) were obtained using BLASTn to search for the common 5′ end consensus in the human genome reference, primarily mapping to chromosomes 5, 11, and 17. Specifically, hits from chromosomes 5 and 11 aligned to the negative strand of the human genome, whereas the main hits from chromosome 17 aligned to the positive strand. Furthermore, the positions of the principal hits closely matched those identified by Bowtie2. The 5′ end consensus was also found on the positive strand of the human mitochondrial genome at position 821, in a region encoding the 12S ribosomal RNA.

**Figure 4.**
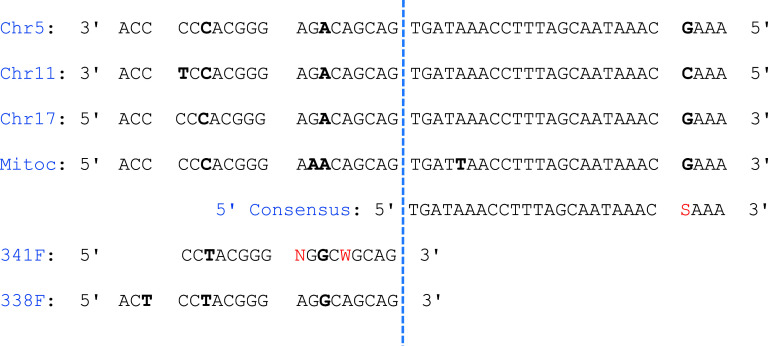
Graphical alignment of the 5′ consensus sequence, primers 338F and 341F, and the corresponding sequences from chromosomes 5, 11, and 17 and the mitochondrion (“Mitoc”). Nucleotides in bold denote mismatches in the alignment. The IUPAC letters in 5′ end consensus and primers are coloured in red. The blue line highlights the position after which the consensus sequence is located on the human chromosomes, excluding the primers sequences.

Initially, no high-identity alignment could be achieved for the 3′ ends of the host-derived sequences. However, when such research was focused on the ASVs derived individually from chromosomes 5, 11, and 17, a consensus for each chromosome was delineated, namely CAGTTTGGGTCTTAGCTATTGTGTGTTCA, CAGTTTGGGTCTTAGTTATTCTGTGTTCA, and CAGTTTGAATCTTCGCTATTGTGTATTCA, respectively (Supplementary Figure S9). These three 3′ end consensus sequences exhibited high similarity to one another but also would require several degeneracies to define a potentially unique 3′ end consensus. In detail, the reverse consensus was found on chromosome 5 on the positive strand at position 81136513, on chromosome 11 on the positive strand at position 10596262, and on chromosome 17 on the negative strand at position 23210708. Notably, the orientation and positions of these sites allow the formation of proper amplicons with the 5′ consensus on each chromosome, yielding products of approximately 250 bp. Furthermore, the common 5′ consensus and the three 3′ consensus sequences were located immediately downstream of regions in the human genome that closely resemble the 338F, 341F, or 805R primers (not included in the reported consensus sequences), with particular similarity to the 3′ ends of each primer. (Figure 4).

Although beyond the main focus of this study, we believe it is noteworthy to report that the 5′ consensus sequence was also identified on the positive strand of *Mus musculus* strain C57BL/10’s chromosome 17 (GenBank accession: OK040662.1) at position 355,000 using BLASTn, with perfect coverage and identity, right after a sequence identical to the one which is before the 5′ consensus in the human chromosome 17.

On the other hand, when the 5′ end consensus sequence was searched in 16S ribosomal RNA sequences of Bacteria and Archaea using BLASTn, imperfect hits were obtained. For instance, the highest-scoring hit was against the positive strand of *Runella rosea* 16S, with a query coverage of 75% (centered around the core of the consensus) and an identity of 94% (considering only the covered portion). Other top hits were related to *Sulforospirillum* and *Thiovibrio*, although these exhibited even lower alignment scores.

### Real-time PCR and electrophoretic run of the off-target amplicons

In the first PCR assay (Supplementary Figure S10), no detectable fluorescence was observed in the negative control by the 25th cycle, while, as expected, the positive control began to fluoresce around the 10th cycle, reaching its plateau by the 21st cycle. In contrast, fluorescence in the human DNA sample appeared after the 20th cycle and did not reach a plateau within 25 cycles. Notably, the positive control exhibited a sharp melting curve peak at approximately 87.5 °C, whereas the human DNA sample showed a broader melting curve ranging from 82 °C to 87.5 °C (Supplementary Figure S10 B).

**Figure 5.**
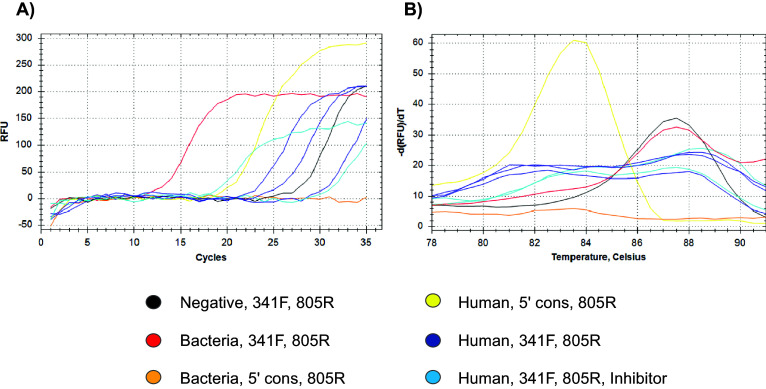
Fluorescence value curves (A) and melting curves (B) of the second PCR samples (listed in Table 2). The colours legend resumes the template type (negative, bacteria, human) and the PCR design (primers and eventual inhibitor). The label “5′ cons” refers to the oligonucleotide designed according to the off-target 5′ consensus and used as forward primer, while “Inhibitor” refers to the same oligonucleotide modified with the 3’ C3-spacer.

Subsequently, a second PCR was performed (as detailed in the methods section and Table 2) with the aim of increasing product yield and visibility (Figure 5). In this second assay, the positive and negative controls confirmed the behaviour observed in the first PCR, although the negative control’s fluorescence curve began to rise after the 25th cycle. The human DNA sample amplified with the 5′ consensus (as forward primer) and the prokaryotic 805R showed fluorescence starting around the 20th cycle and reached a plateau with a higher fluorescence signal than the other samples, while the faecal DNA (mainly bacterial), amplified through the same primer pairs, showed no detectable fluorescence, even by the 35th cycle. Technical replicates of the human DNA PCR showed variable fluorescence onset between cycles 23 and 30. The two reactions using human DNA, the prokaryotic primer pair, and the off-target inhibitor at 0.25 μM and 0.5 μM concentration, began to fluoresce at cycles 30 and 20, respectively, although their plateau fluorescence levels were lower.

The melting curves of both the positive and negative controls were similar, while those of the human DNA amplified with prokaryotic primers were broad, as previously observed (Figure 5B). Moreover, the melting curve of the human DNA amplified with the 5′ off-target consensus and the 805R primer showed a distinct peak between 83 °C and 84 °C (Figure 5B).

Finally, amplicon sizes were checked by electrophoresis (Supplementary Figure S11). Both the negative and positive controls showed bands between 450 and 500 bp, consistent with the expected V3-V4 16S region, with the positive control band appearing noticeably more intense. All reactions involving human DNA yielded about 300 bp bands, corresponding to the expected size of the main off-targets (including primers in the off-target length computation). Specifically, the human DNA amplified with the 5′ consensus as forward primer showed a single clear band, while the sample amplified with both the prokaryotic primer pairs exhibited multiple bands of the same size. The technical replicates showed variable band intensities, despite being aliquots of the same master mix. The two reactions containing the off-target inhibitor displayed different behaviours: the 0.5 μM inhibitor sample showed an amplicon with the same length as that of the other human DNA involving reactions, along with another band compatible with the 16S V3-V4 amplicon, whereas the 0.25 μM inhibitor sample lacked a visible 16S band.

The third PCR confirmed the results of the second PCR, showing that human off-target amplification decreased as the inhibitor concentration increased, while allowing the kitome sequences to become more prominent (Supplementary Figures 12 and 13).

## Discussion

Microbiome research employing molecular ecology-based approaches remains a relatively young field, as the advent of next-generation sequencing (NGS) technologies catalysed its rapid expansion only approximately two decades ago (Slatko et al., 2018). Nevertheless, our understanding of human-associated microbial symbionts has progressively improved due to the collective contributions of researchers globally. In addition to studies profiling the microbiota, several investigations have elucidated the methodological constraints of this field, identifying potential sources of false positives and corresponding mitigation strategies (Vestheim & Jarman, 2008; Bharti & Grimm, 2019; Cao et al., 2020; Walker et al., 2020; Di Gloria et al., 2024). For instance, both ubiquitous contaminants in reagents used for DNA-to-amplicon preparation (termed “kitome”) and host off-targets are, in our opinion, rather unexpected and poorly understood issues to emerge from sequencing yet they are consistently observable in analyses of tissues with a low microbe-to-host DNA ratio (Paniagua Voirol et al., 2021; Di Gloria et al., 2024). To contribute to potential solutions, we explored the generation of human off-targets to shed light on the mechanisms behind these occurrences.

Initially, we observed that the amount of off-target sequences varied markedly both across bioprojects and between samples within a given bioproject, ranging from 0.1% to 30% of total reads. This variability suggests a significant stochastic component in the formation of off-targets. However, the pronounced off-target contamination observed in specific bioprojects may also be attributable to deterministic factors, such as the abundance of host DNA in the original sample or the particular extraction and amplification kits and reagents, as well as the PCR kit used. Unfortunately, validating this hypothesis remains challenging, as the precise combination of kits used is rarely disclosed in the papers. Regardless, variability in off-target frequency among samples within the same bioproject suggests that, even when using specific library preparation kits, random factors may still play a major and uncontrollable role in off-target generation.

When off-target sequences predominate, key results may be obscured, and even entire samples may be excluded due to inadequate ecological coverage. For example, 25 samples in the present study were excluded from ecological analyses based on rarefaction curve criteria. Of these, 23 originated from a bioproject with a high proportion of off-targets, suggesting that their lack of saturation likely resulted from sequencing depth being exhausted by non-target amplicons. Notably, this limitation cannot be circumvented by in silico decontamination of off-target reads after sequencing. Further complicating this picture, the unevenness in remaining library size (i.e., total read abundance) after decontamination is aggravated by the aforementioned variability in off-target occurrence, which in turn hampers statistical power in detecting ecological differences (Schloss, 2024). Although certain transformations, such as rarefaction, have been shown to mitigate issues associated with large disparities in library size (Schloss, 2024), which transformation is the most methodologically sound remains a matter of ongoing debate in literature.

Furthermore, off-targets influenced the performance of the taxonomic scikit-learn classifier implemented in QIIME2, which would have led to an increased number of unclassified or misclassified bacterial sequences if left unnoticed. This issue arises because the classifier analyses the first 100 sequences in the dataset by default and may infer reverse (or, in any case, wrong) read orientation if classification fails in the expected direction, which is particularly likely when host sequences are present. If not recognized and properly addressed, such automatic inference can lead to misclassification of entire bioprojects, ultimately resulting in highly misleading outcomes. Importantly, even after manual correction of read orientation, the off-targets contributed to a slight increase in sample dispersion (as measured by ecological distances) and led to the false detection of microbial taxa not present in the dataset, while simultaneously masking true biologically significant patterns. The misidentification of a small number of human sequences is expected, as the database used to train the classifier contains only a specific subset of sequences. As a result, false positives can occur when sufficient similarity is detected between the query sequence and such a subset (Di Gloria et al., 2025). For example, few human sequences were identified as protozoan using the Bayesian classifier trained on SILVA 138 SSU database (mainly designed for microbial taxonomy), as organisms that appear highly different from a traditional human-centric perspective can actually be quite similar when viewed through the lens of the broader prokaryote–eukaryote distinction. Using a more comprehensive database could mitigate this issue, but at the cost of a likely reduction in sensitivity for the classification of true prokaryotic reads (Di Gloria et al., 2025). Moreover, in the context of 16S amplicon sequencing, including sequences unrelated to the small ribosomal subunit rRNA would be conceptually inappropriate within the workflow design.

Despite these limitations, the ecological findings of this meta-analysis are consistent with many previously published observations of the human gut microbiota. These include the predominance of certain taxa (as detailed in Table 3 and Figure 1) (Piquer-Esteban et al., 2022; Deissová et al., 2023), as well as the differential abundance of *Fusobacterium*, *Blautia*, and *Campylobacter* in CRC tissues compared to healthy controls (Rubinstein et al., 2013; Costa et al., 2022; Russo et al., 2023). However, these differential abundances would not have reached statistical significance if host-derived sequences had been retained, likely due to increased noise introduced during the abundance transformation performed by DESeq2. In this regard, it is noteworthy that different data transformations and differential abundance algorithms are expected to yield different outcomes and to be influenced differently by noises such as that derived by the off-targets (Nearing et al., 2022), however, a comparison of bioinformatic analysis approaches falls outside the scope of this article.

Following our examination of the biases introduced by off-targets, we investigated their origins and identified recurrent ASVs aligning to specific regions of the human chromosomes 5, 11 and 17. Specifically, the consensus sequence TGATAAACCTTTAGCAATAAACSAAAGTTTAA was found at the 5′ end of ASVs derived from these regions. Similar results were obtained from three additional bioprojects involving human tissues other than the colon, indicating that these outcomes are not tissue-specific.

A sequence with high similarity to the prokaryotic 341F primer was found upstream of this consensus region on either the positive or negative DNA strand, and a corresponding sequence resembling the 805R primer was identified on the opposing strand within a fragment length (about 300 bp) suitable with the NGS sequencing albeit being shorter than the typical V3–V4 amplicon (about 460 bp). The involvement of these human genome segments was further validated through the second PCR assay we performed, wherein a PCR on human genome using a forward primer designed on the human consensus sequence paired with 805R produced an amplicon comparable in size to that produced by 341F/805R (Figure 5A,B and Supplementary Figure S11), as predicted. This human forward primer did not produce “off–off-target” amplicons when tested on a bacteria-rich matrix, confirming its specificity to the human genome. These recurrent off-target amplicons exhibited a melting point of approximately 82.5 °C - 84 °C in qPCR assays and consistently amplified at much later cycles than the bacterial DNA, as expected. Nevertheless, the three identified genomic regions were not the sole sources of off-target amplification, as several ASVs aligned to other less recurrent loci, reinforcing the conclusion that stochasticity contributes to off-target generation. The variable off-target occurrence between and within bioprojects further supports the hypothesis of their random generation.

However, the presence of recurrent off-target sequences suggests that the roll of the dice behind off-target amplification is nonetheless biased toward certain genomic regions due to inherent biological factors.

Indeed, the alignment data in Figure 4 reveal that the 341F and 805R annealing on these recurrent regions requires few mismatches in the 5′ or central regions of the primer, thereby allowing a perfect match of the 3′ region, which is rich in GC content. Such mis-priming conditions are well-documented in site-directed mutagenesis experiments (Kwok et al., 1990), the observed recurrences are biologically plausible.

Accordingly, we tried to address the off-target issue by using a 3′-spacer-modified oligonucleotide designed on the human consensus sequence defined above. This inhibitor was effective even at concentrations half that of the primers and demonstrated improved suppression of off-target amplification at higher concentrations. Although kitome-related amplicons became more evident in the absence of human off-targets following the inhibition (Figure 5B, Supplementary Figure S11, Supplementary Figure S12 B, Supplementary Figure S13), bacterial DNA from actual biological samples would be clearly favoured over kitome contaminants. It is important to note that prokaryotic kitome amplicons only became visible after 25 PCR cycles, whereas off-targets were detectable even before the standard 25 cycles used in the Illumina protocol (Supplementary Figure S10), despite employing a primer pair designed for prokaryotes. However, the melting curve associated with these amplicons suggests that kitome may have partially contributed to the fluorescence signal observed in the human DNA sample (Supplementary Figure S10 B).

Based on our findings, we propose the following approaches for mitigating off-target amplification in human colon-sourced samples, whereas the V3-V4 16S primers are employed:

1. Inhibiting most of the off-target amplification using a modified oligonucleotide as an inhibitor targeting the most recurrent human sequences we identified;
2. Employing a preliminary qPCR to monitor the amplification behaviour of a small subset of study samples along with high microbial biomass samples to determine the optimal number of PCR cycles for library preparation, leveraging the preferential amplification of bacterial sequences at lower cycle, although this approach deviates from the standard Illumina 16S (n.d.) workflow;
3. Isolating the 16S-specific band based on size differences from off-target products, as most of the off-targets are far shorter than the V3–V4 amplicon, although this solution may be impractical for analysis involving numerous samples and could introduce additional contamination risks.

We argue that the inhibition of off-target generation represents the most feasible and effective mitigation strategy among those proposed here. This approach directly addresses the root cause of the problem, preserving sequencing efficiency, minimizing loss of statistical power, and, most importantly, preventing the potential depletion of sequencing depth due to off-target amplicons. Notably, the inclusion of an oligonucleotide in PCR reactions represents a relatively simple and cost-effective approach that, according to our results, is not expected to introduce biases when specifically directed against the reported 5′ consensus sequence. However, given the stochastic nature of off-target generation and the broad melting curve in the PCR involving human DNA, we expect that off-targets from other (less probable) regions may also emerge, albeit rarely, and implementing an inhibitor for each potential source region could be impractical or even deleterious. Furthermore, it is worth noting that the proposed inhibitor developed and tested in this study still requires further validation, such as dedicated benchmarking including alternative inhibitors, using known mock communities as positive controls, and testing also on other organisms such as *M. musculus*, which is often used as a model in place of human subjects. Nevertheless, the primary objective of this study was to elucidate the reasons underlying off-target generation and, in this regard, the proposed inhibitor served as evidence supporting the accuracy of the consensus sequence we identified, paving the way to future research. In addition, our results underscore the significant and risky off-target biases in samples with a high host-to-bacteria DNA ratio, which are still often underestimated or even overlooked in current literature.

## Supporting information

Di Gloria et al. supplementary material Di Gloria et al. supplementary material

## Data Availability

All sequences analysed in this study are publicly available on the NCBI SRA under the accession codes listed in Table 1. The scripts, the processed data and further details are accessible via the GitHub repository referenced in the Methods section.

## Abbreviations

ASV: Amplicon Sequencing Variant
Consensus sequence: sequence motif recurring across multiple sequences
CRC: Colon Rectal Cancer
IBD: Intestinal Bowel Disease
Kitome: ubiquitous DNA sequences in DNA extraction and/or PCR kits
Mis-priming: primer annealing to non-perfectly identical sequences on the template
NGS: Next-Generation Sequencing
Off-targets: amplicon obtained despite improper primer annealing to the template
PCoA: Principal Coordinate Analysis

## Supplementary material

The supplementary material for this article can be found at http://doi.org/10.1017/gmb.2025.10012.
