# Supplementary material for: Human DNA hijacking microbiota surveys: causes and consequences in colon related 16s rRNA amplicon sequencing
Source: Gut Microbiome (Camb). 2025 Aug 19;6:e14. doi: 10.1017/gmb.2025.10012 (PMC12455517; doi:10.1017/gmb.2025.10012)
Supplement: Di Gloria et al. supplementary material [file S2632289725100121sup001.zip › Supplementary figure 9 _ reverse consensus _ caption.pdf]

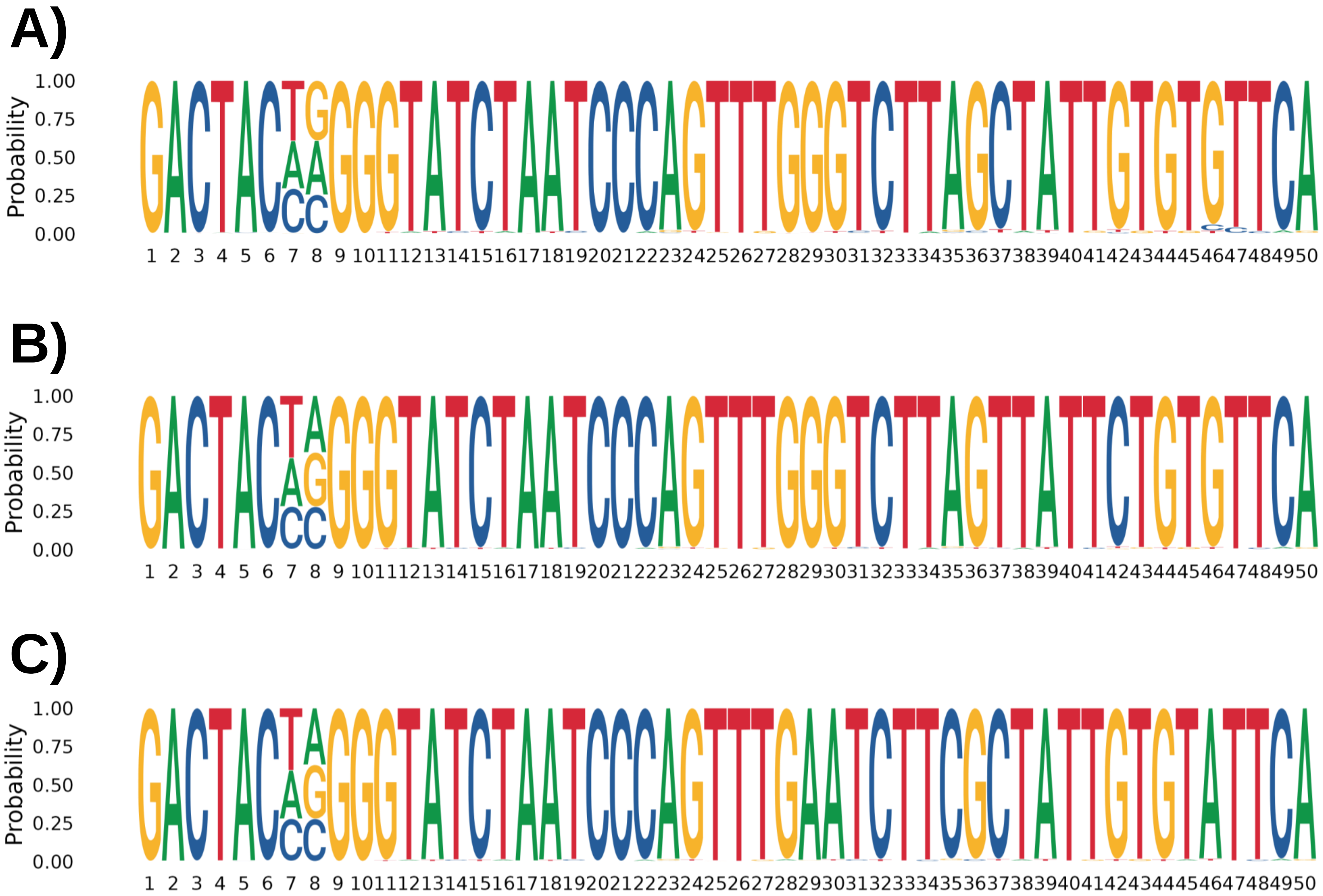
A)
B)
C)
Probability
1.00
0.75
0.50
0.25
0.00
