# Supplementary figures and images for: Human DNA hijacking microbiota surveys: causes and consequences in colon related 16s rRNA amplicon sequencing
Source: Gut Microbiome (Camb). 2025 Aug 19;6:e14. doi: 10.1017/gmb.2025.10012 (PMC12455517; doi:10.1017/gmb.2025.10012)

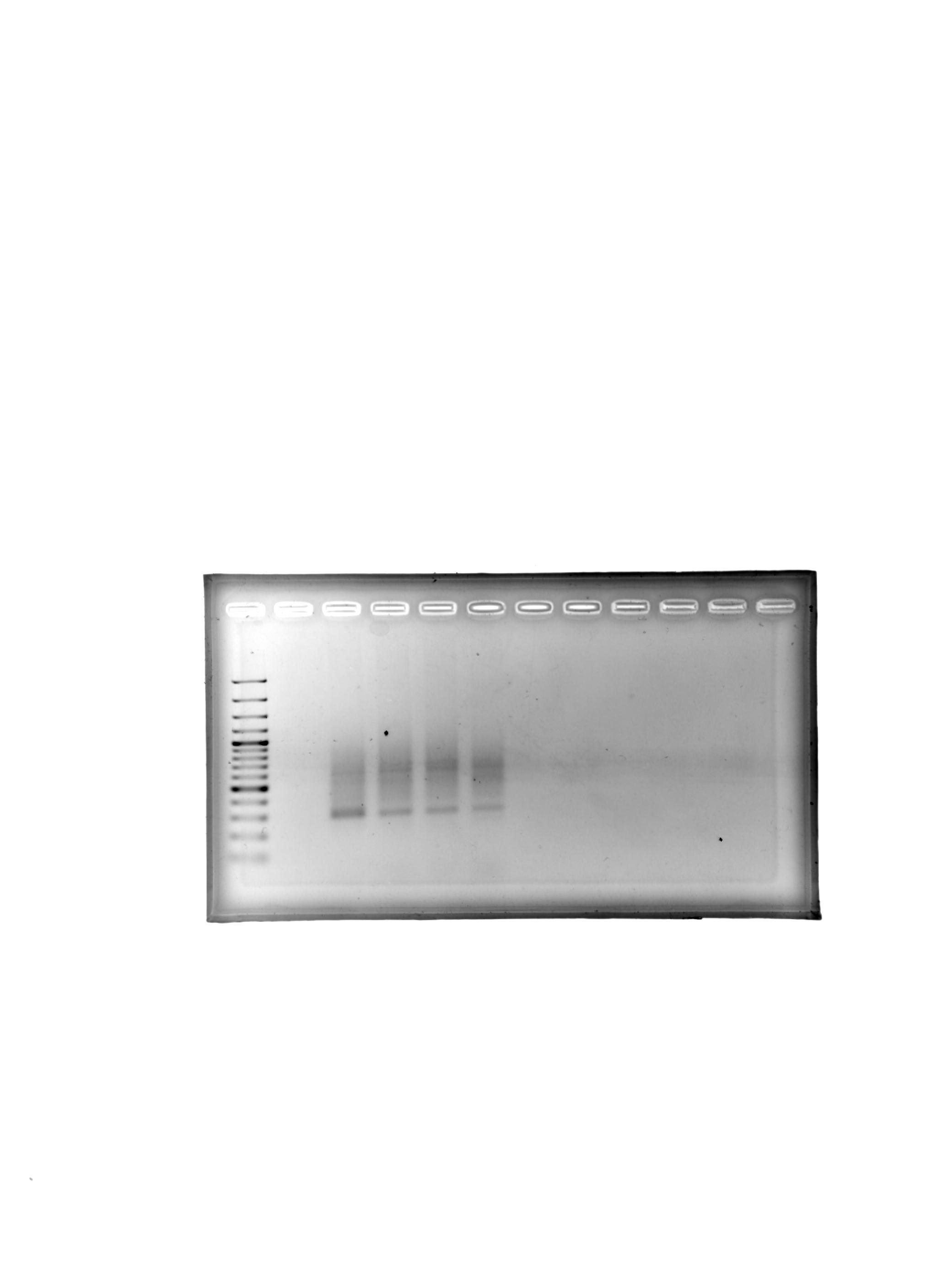

Supplement: Di Gloria et al. supplementary material [file S2632289725100121sup001.zip › Supplementary figure 13 _ gel third qPCR _ caption.jpg]

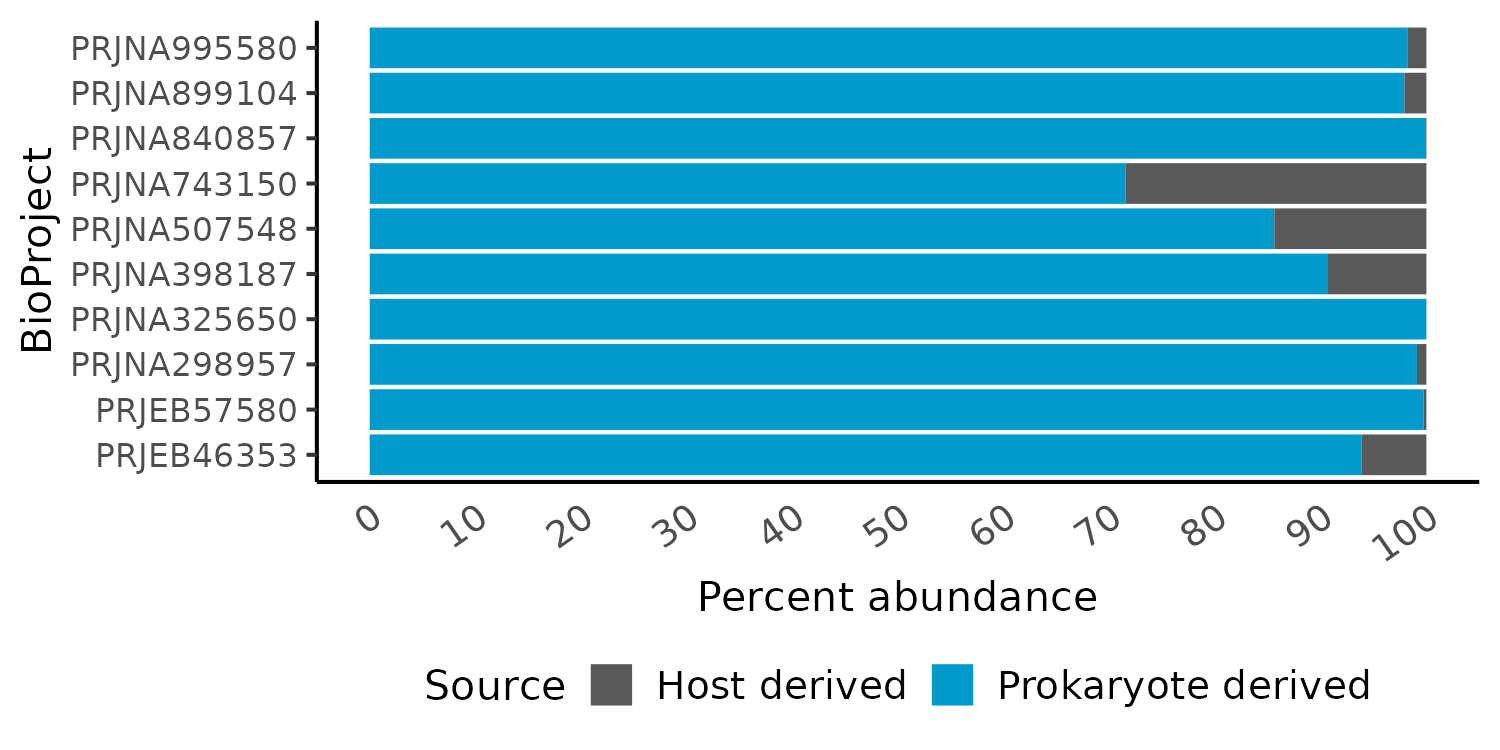

Supplement: Di Gloria et al. supplementary material [file S2632289725100121sup001.zip › Supplementary Figure 1 _ caption.png]

**A)**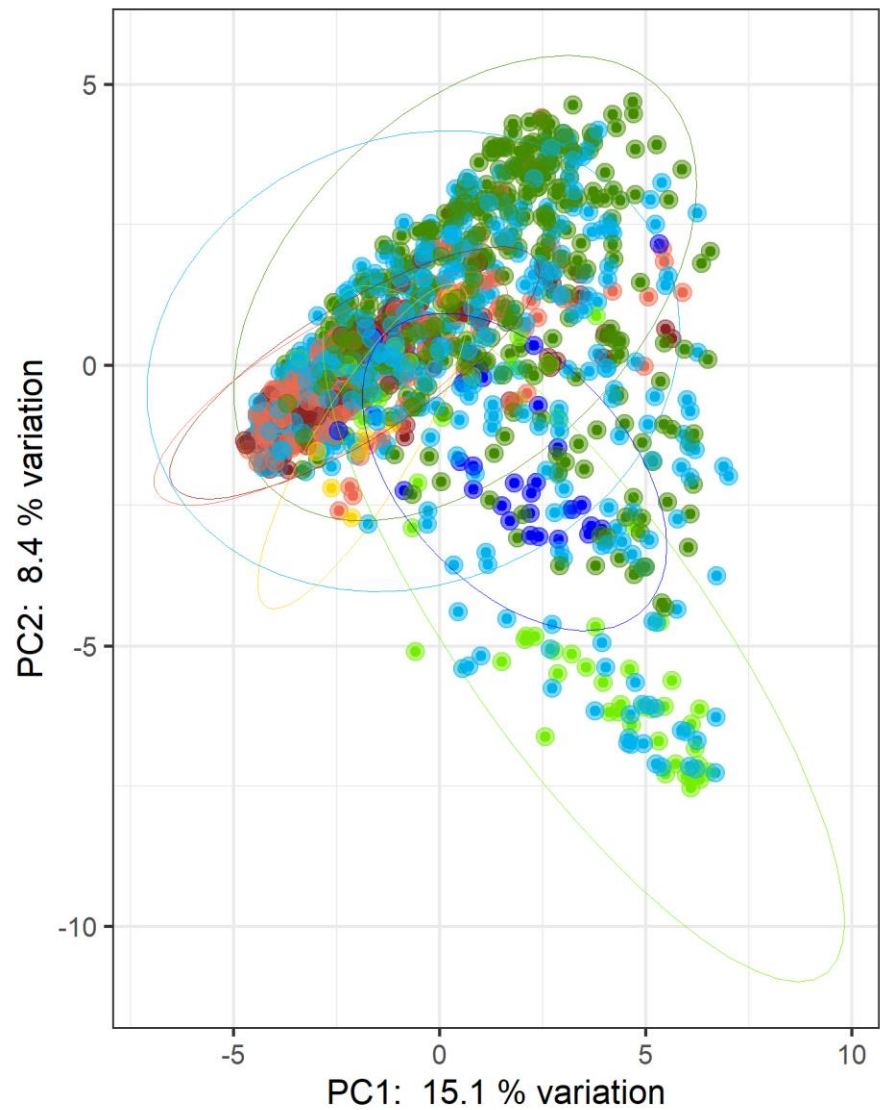**B)**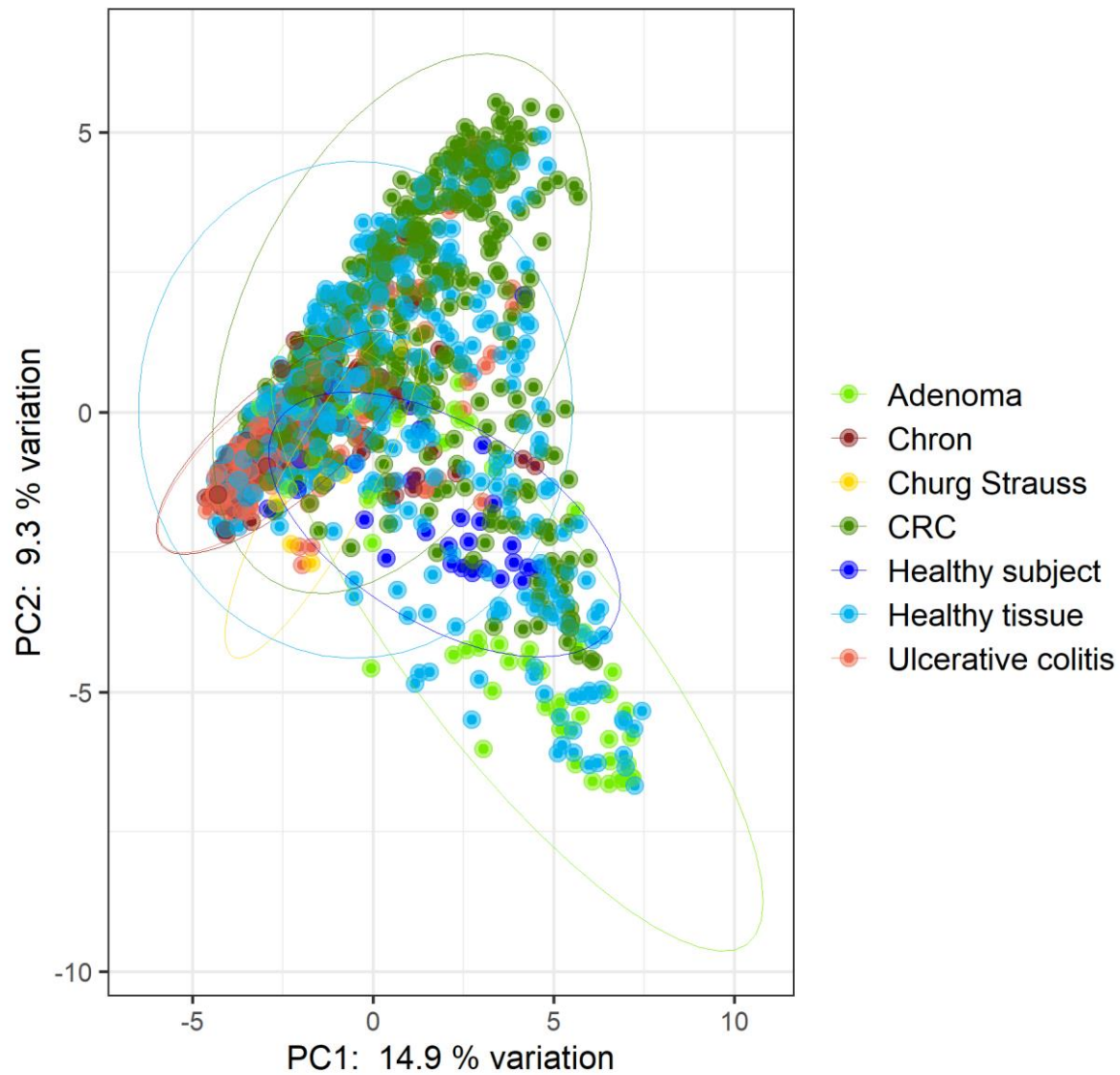

Supplement: Di Gloria et al. supplementary material [file S2632289725100121sup001.zip › Supplementary figure 2 _ PCoA status _ caption.pdf]

**A)**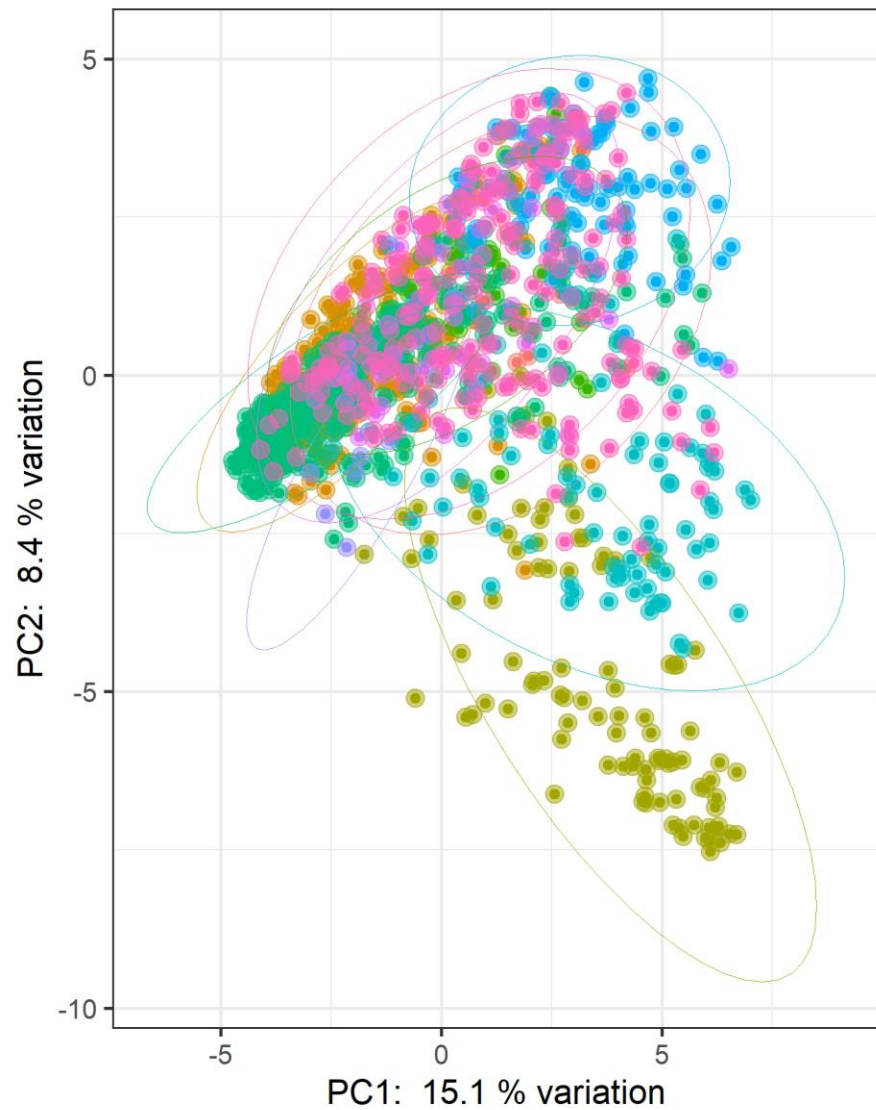**B)**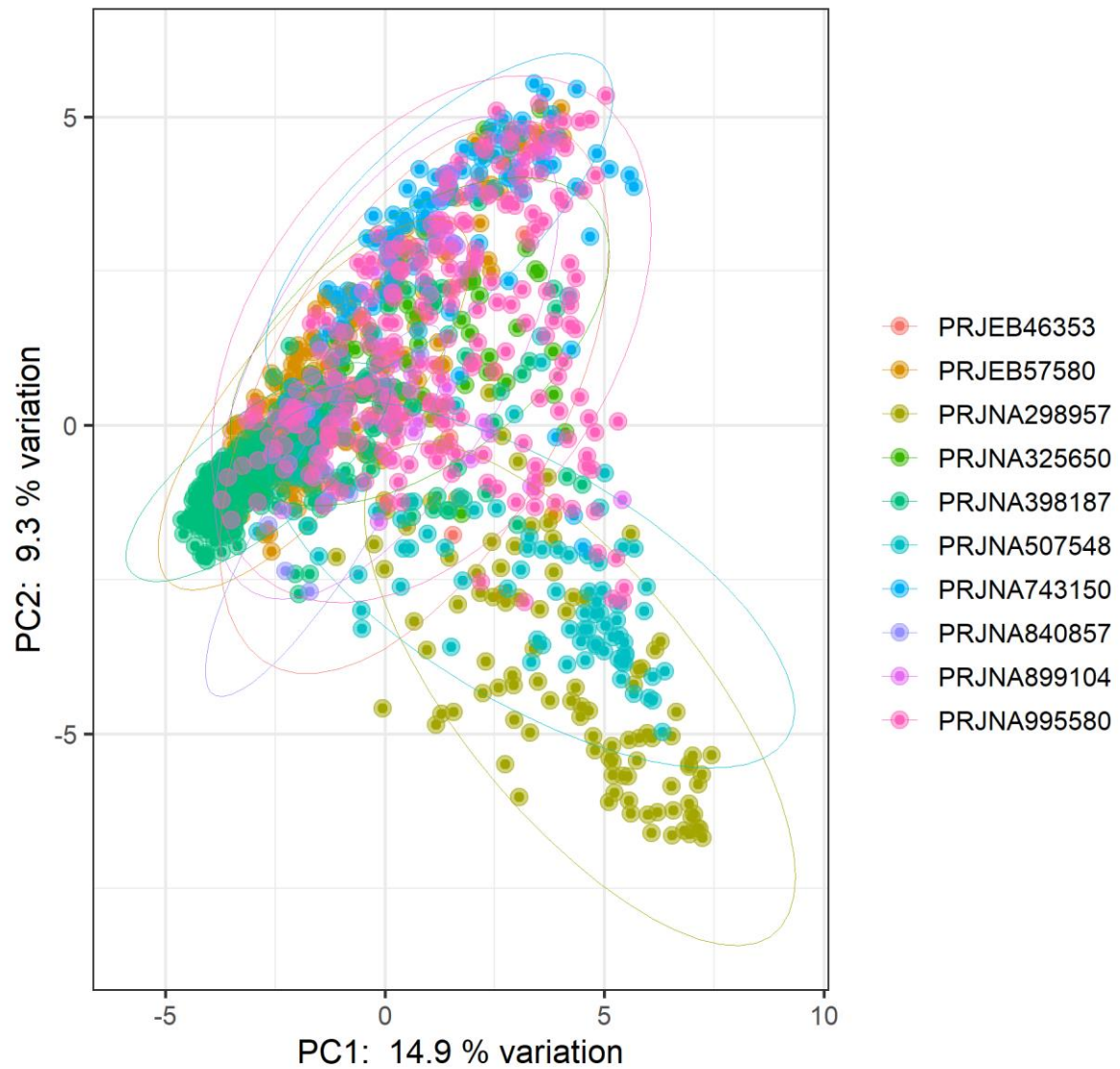

Supplement: Di Gloria et al. supplementary material [file S2632289725100121sup001.zip › Supplementary figure 3 _ PCoA project _ caption.pdf]

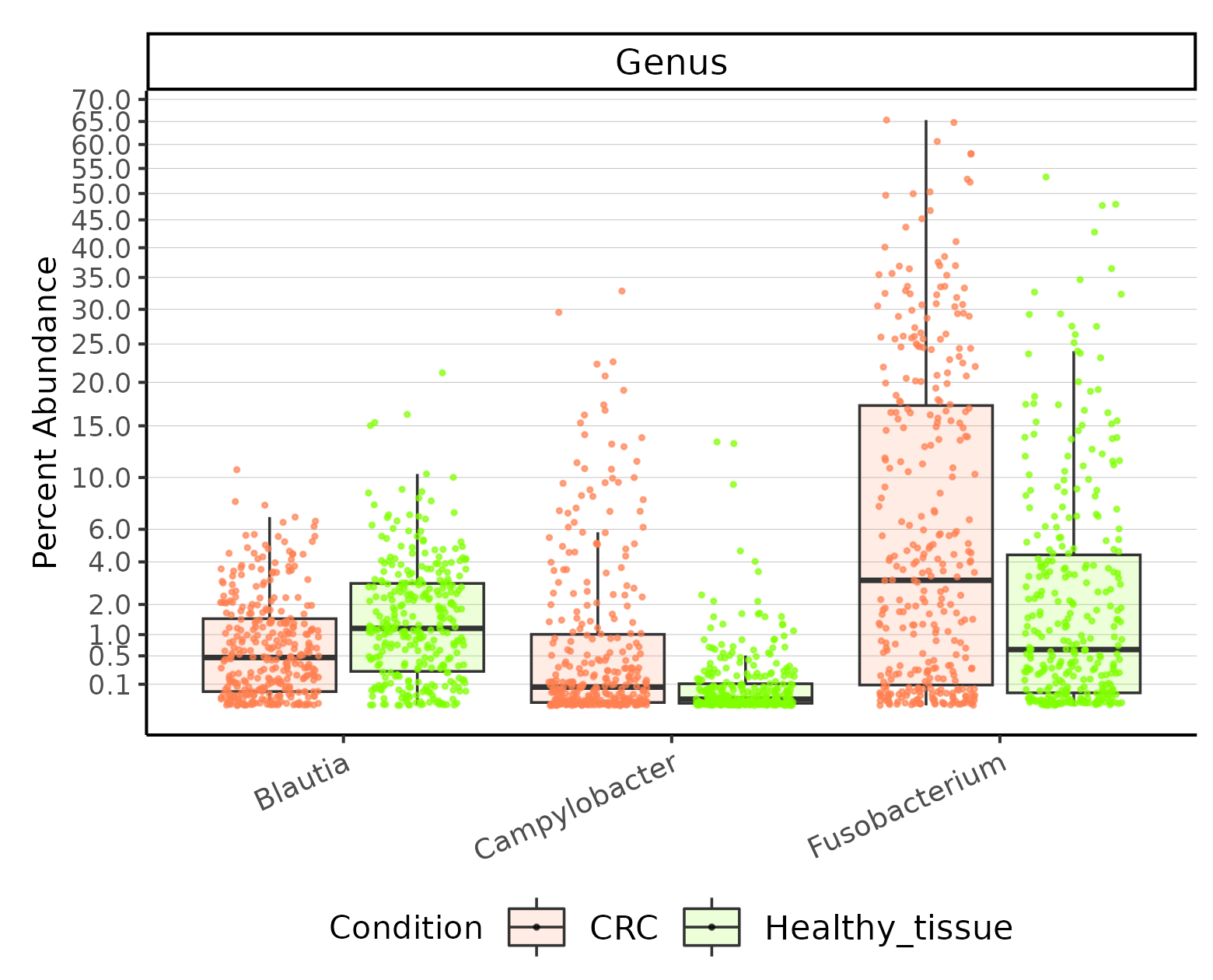

Supplement: Di Gloria et al. supplementary material [file S2632289725100121sup001.zip › Supplementary figure 4 _ DESeq2 _ caption.png]

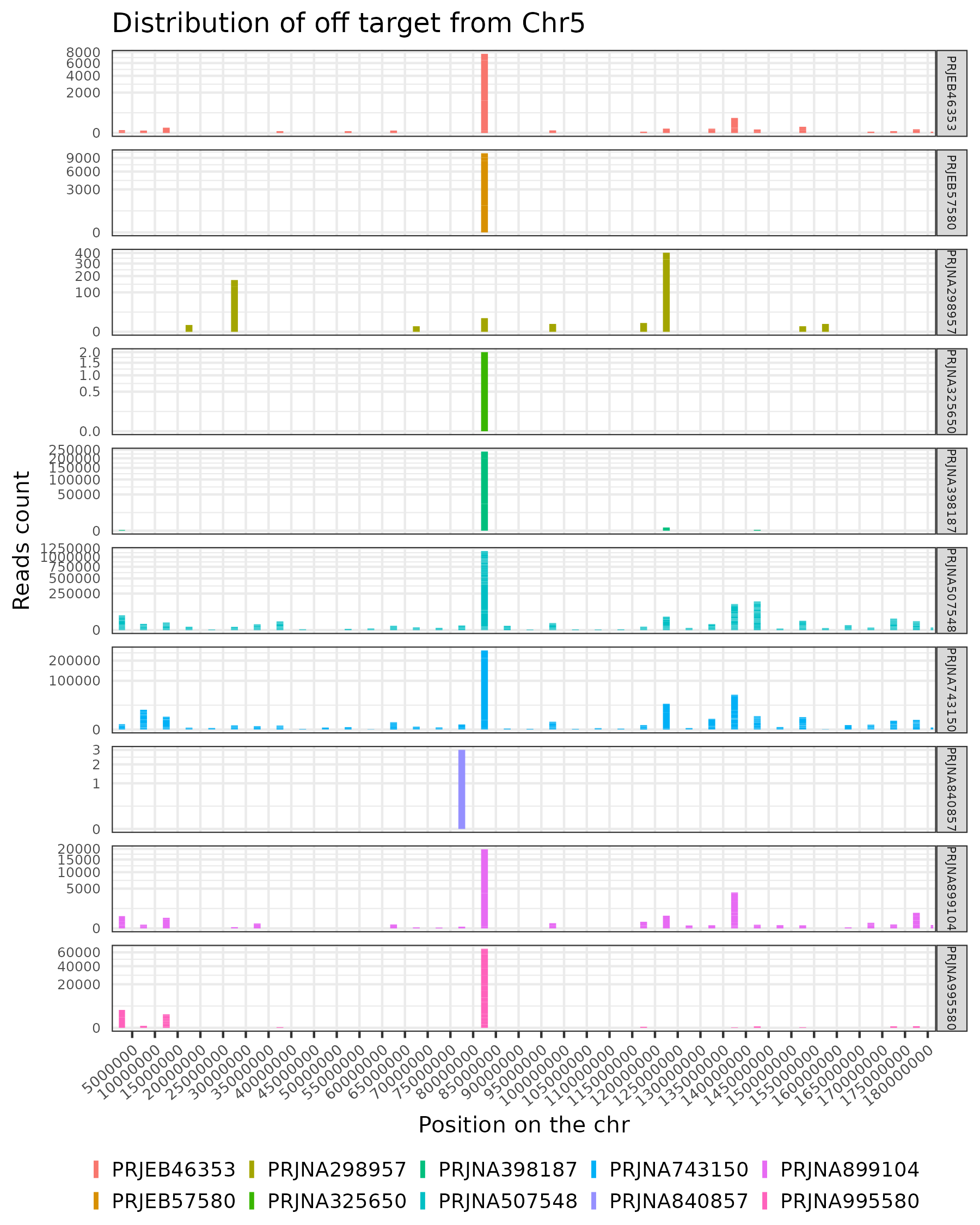

Supplement: Di Gloria et al. supplementary material [file S2632289725100121sup001.zip › Supplementary figure 5 _ Chr5 _ caption.png]

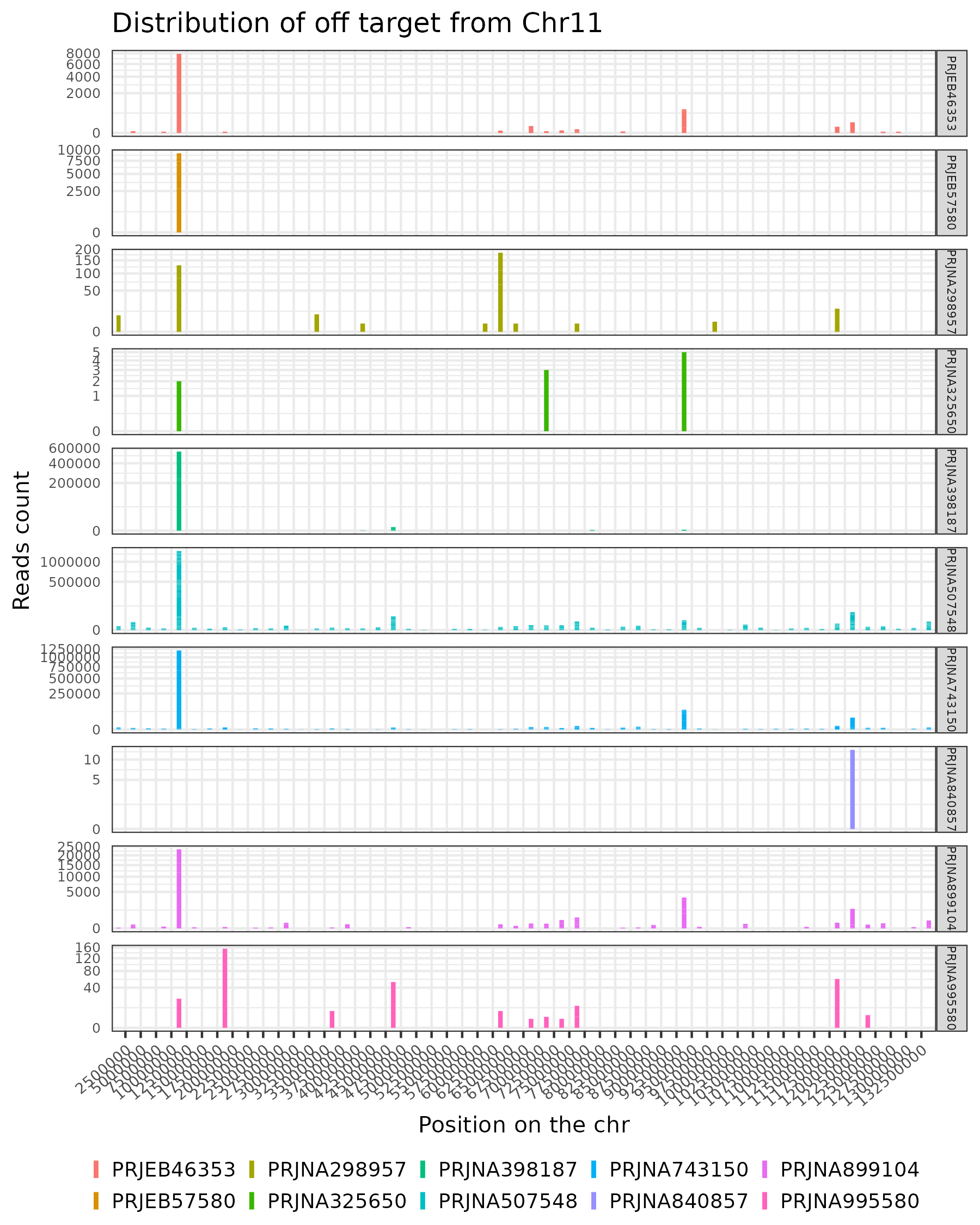

Supplement: Di Gloria et al. supplementary material [file S2632289725100121sup001.zip › Supplementary figure 6 _ Chr11 _ caption.png]

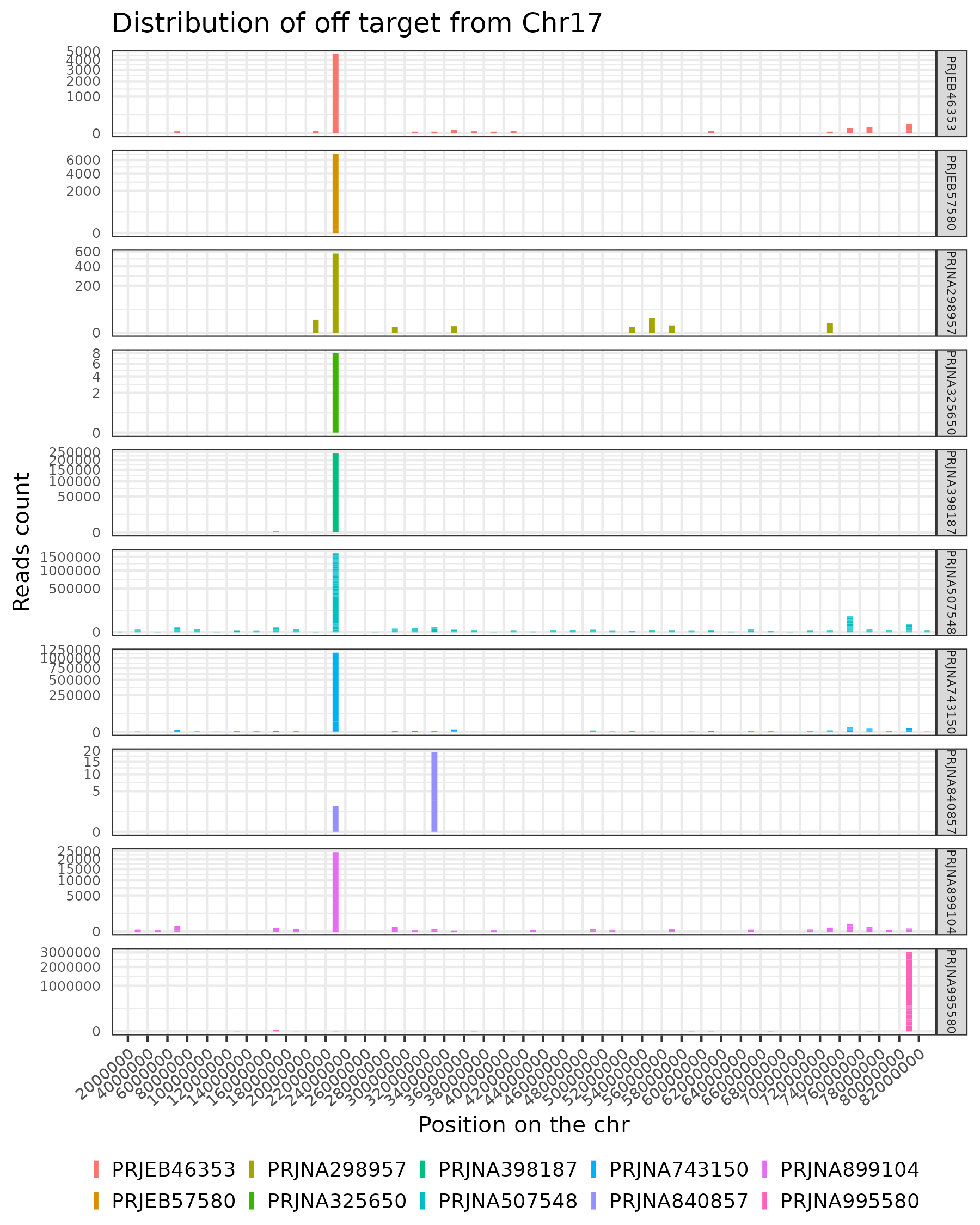

Supplement: Di Gloria et al. supplementary material [file S2632289725100121sup001.zip › Supplementary figure 7 _ Chr17 _ caption.png]

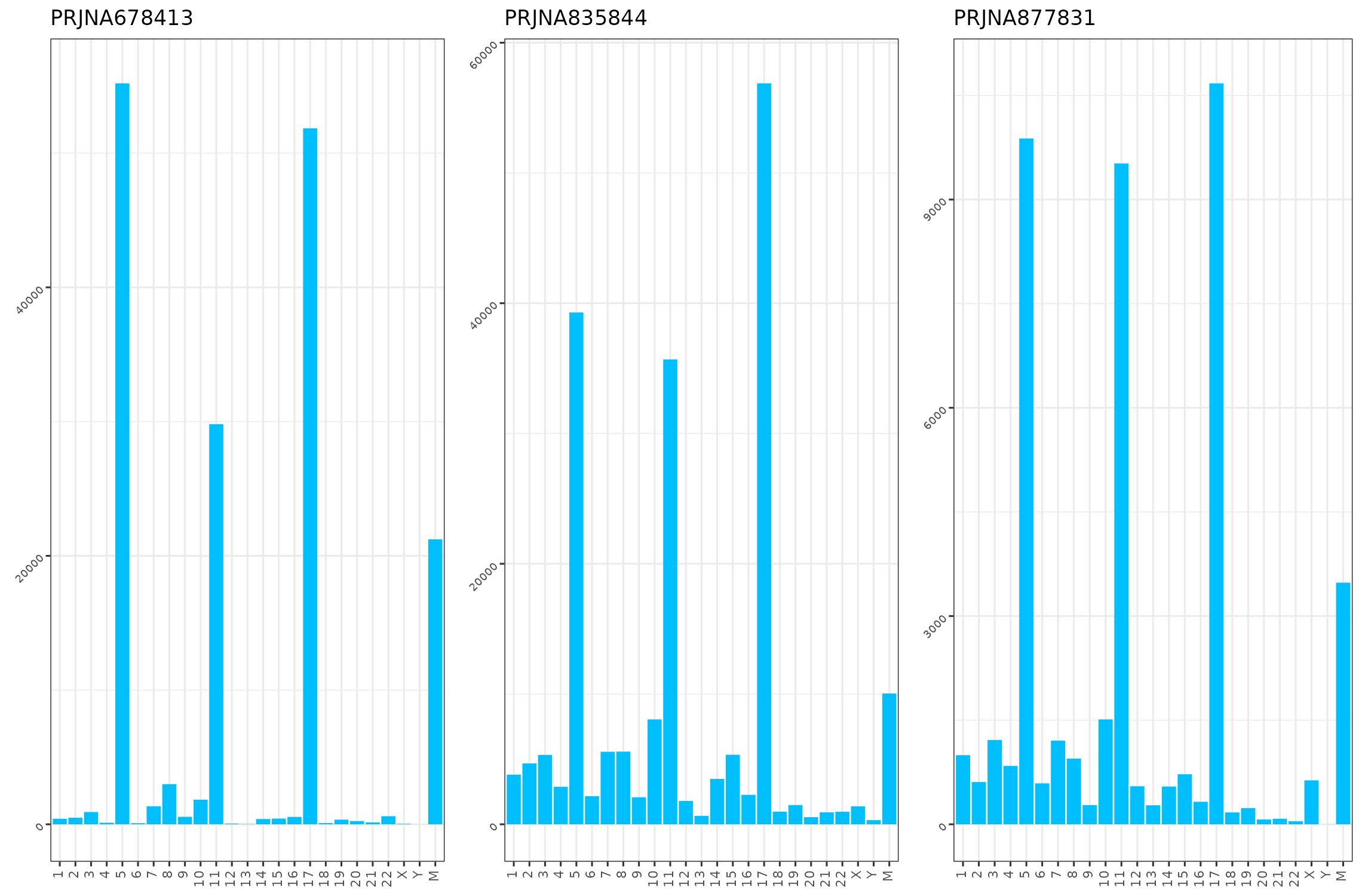

Supplement: Di Gloria et al. supplementary material [file S2632289725100121sup001.zip › Supplementary figure 8 _ EXTRA non colon _ caption.png]

**A)**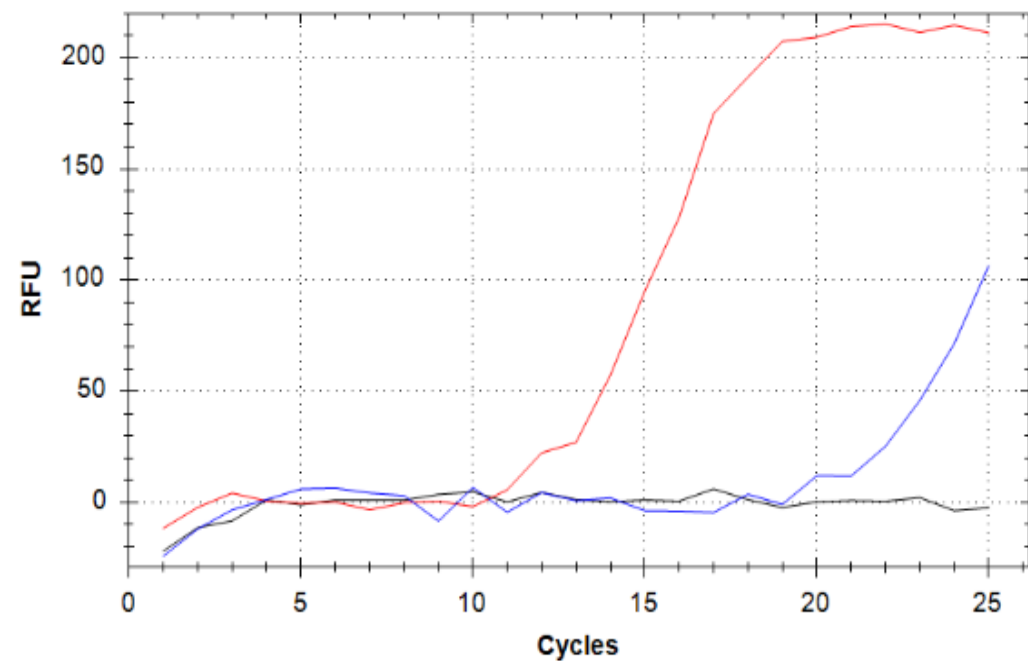

● Negative, 341F, 805R

**B)**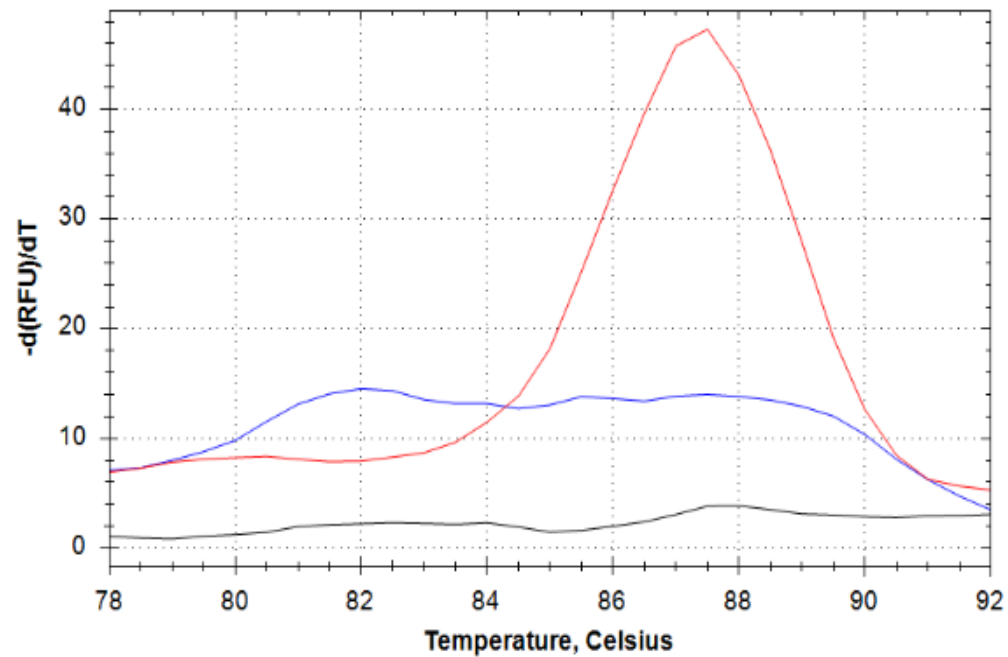

● Bacteria, 341F, 805R

● Human, 341F, 805R

Supplement: Di Gloria et al. supplementary material [file S2632289725100121sup001.zip › Supplementary figure 10_ first qPCR _ caption.pdf]

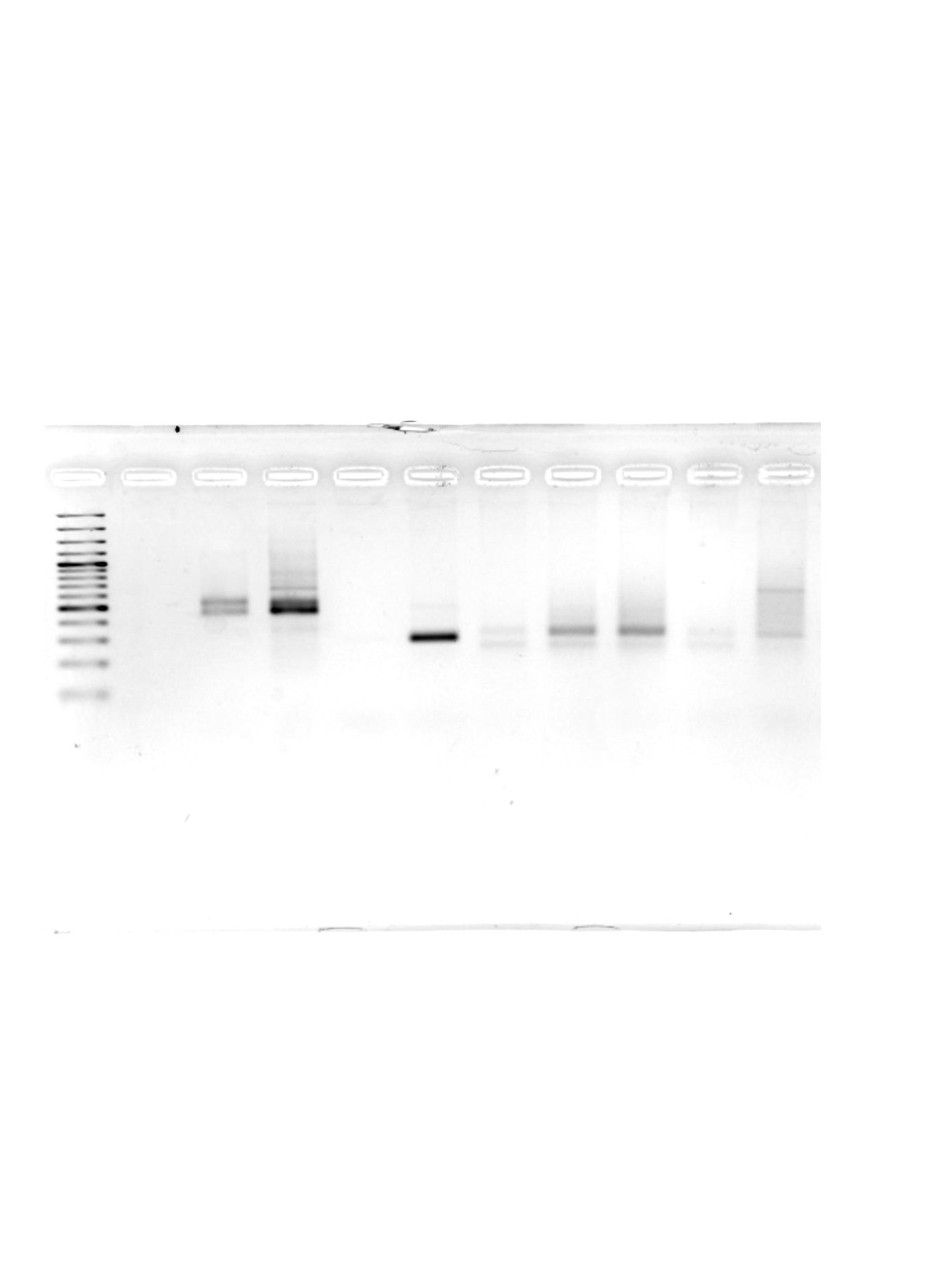

Supplement: Di Gloria et al. supplementary material [file S2632289725100121sup001.zip › Supplementary figure 11_ gel second qPCR _ caption.jpg]

**A)**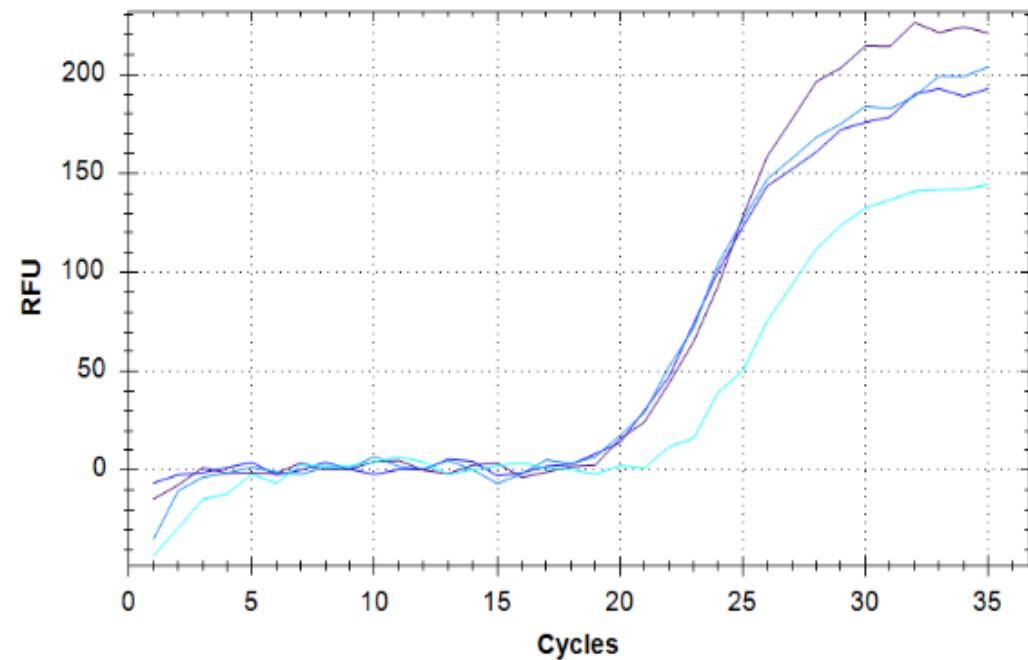

0 M

0.25 M

**B)**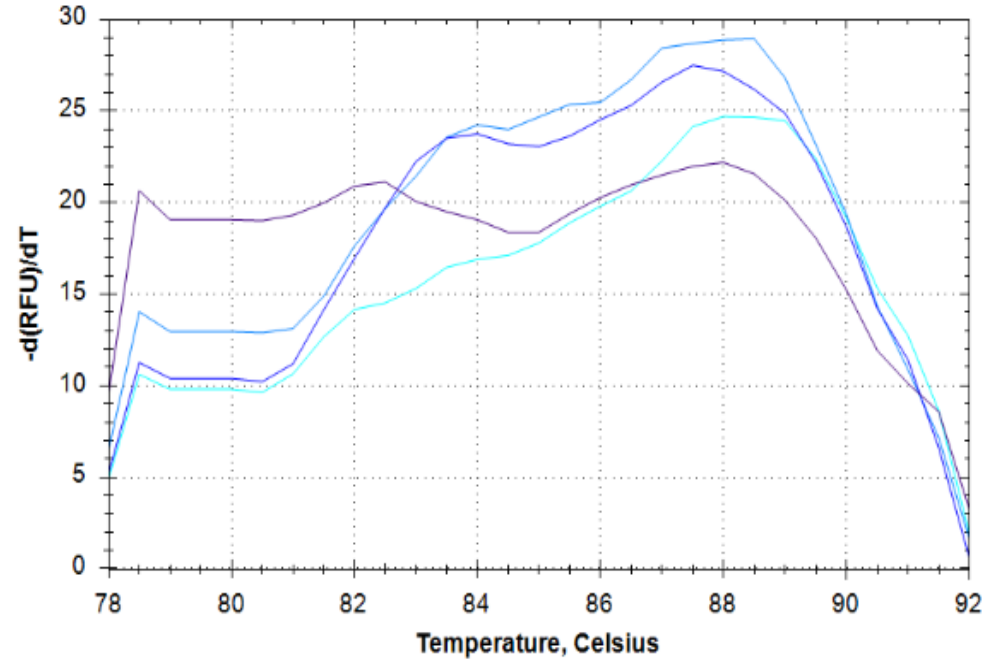

0.5 M

1 M

Supplement: Di Gloria et al. supplementary material [file S2632289725100121sup001.zip › Supplementary figure 12_ third qPCR _ caption.pdf]
